# Changes in the Metabolome of Different Tissues in Response to Streptozotocin Diabetes and Mildronate Exposure: A Metabolomic Assessment

**DOI:** 10.3390/metabo16010061

**Published:** 2026-01-09

**Authors:** David Hauton, Dragana Savic, John Walsby-Tickle, Damian Tyler, James S. O. McCullagh

**Affiliations:** 1 Chemistry Research Laboratory, Metabolomics Research Group, Department of Chemistry, University of Oxford, Mansfield Road, Oxford OX1 3TA, UK; 2 Department of Physiology, Anatomy & Genetics, University of Oxford, Parks Road, Oxford OX1 3PT, UK; 3 Faculty of Life Sciences, University of Bath, Claverton Down, Bath BA2 7AY, UK; 4 Oxford Centre for Clinical Magnetic Resonance (OCCMR), Division of Cardiovascular Medicine, Radcliffe Department of Medicine, University of Oxford, Oxford OX3 9DU, UK

**Keywords:** Steptozotocin diabetes, mildronate, brain, liver, plasma, metabolomics, hyperglycaemia

## Abstract

**Background:** Uncontrolled diabetes is characterised by a loss of blood glucose control and increased oxidation of fatty acids to produce ATP. Use of metabolic inhibitors to blunt fatty acid oxidation and restore glucose metabolism is a poorly studied intervention for diabetes. **Methods**: Steptozotocin-induced diabetes was developed in Wistar male rats. A subset was supplemented with mildronate (100 mg/kg—14 days). Exploiting liquid chromatography-mass spectrometry for workflows including ion exchange-, C18-reverse phase- and HILIC-based chromatography methods, metabolite levels were quantified in plasma liver and brain tissue. Using both untargeted and targeted metabolomic analysis changes to the global tissue metabolome and individual metabolic pathways were estimated. **Results**: We document that an inhibitor of carnitine synthesis, mildronate, decreased plasma (50% *p* < 0.01) carnitine abundance and decreased plasma glucose concentration by one-third compared to streptozotocin (STZ)-treated rats (*p* < 0.001). Targeted metabolomic analysis of the liver showed decreased alpha-ketoglutarate abundance (35% *p* < 0.05) by STZ diabetes that was further decreased following mildronate treatment (50% *p* < 0.05). For both beta-hydroxybutyrate and succinate levels, STZ diabetes increased hepatic abundance by 50% (*p* < 0.05 for both), which was restored to control levels by mildronate (*p* < 0.05 for both). In contrast, brain TCA intermediate abundances were unaffected by either STZ diabetes or mildronate (NS for all). STZ diabetes also decreased abundance of pentose phosphate pathway (PPP) metabolites in the liver (glucose-6-phosphate, 6-phosphogluconolactone, 6-phosphogluconate 50% for all; *p* < 0.05), which was not restored by mildronate treatment. However, brain PPP metabolite abundance was unchanged by STZ diabetes or mildronate (NS for all). However, mildronate treatment did not affect the increased abundance of brain sorbitol, sorbitol-6-phosphate and glucose-6-phosphate as a result of STZ diabetes. **Conclusions**: Together, these observations highlight the potential role that metabolic inhibitors, like mildronate, may play in restoring blood glucose for diabetic patients, without a direct effect of tissues that represent obligate consumers of glucose (e.g., brain) whilst manipulating fat oxidation in tissues such as the liver.

## 1. Introduction

Diabetes represents a chronic metabolic disease, characterised by either an absolute loss of the production of insulin (Type I Diabetes—insulin-dependent diabetes) [1] or a decrease in sensitivity to insulin (Type II diabetes—non-insulin-dependent diabetes) [2]. As a consequence, plasma glucose concentrations are elevated, reflecting the combination of aberrant control of blood glucose from ingested glucose and increased hepatic gluconeogenesis [3]. In addition, further effects of aberrant control of blood glucose are also accompanied by elevated fatty acids and triacylglycerol through removal of the inhibitory effect of insulin on hormone-sensitive lipase, driving increased lipolysis [4].

For uncontrolled diabetes, this dysregulation of plasma glucose concentrations mediates alterations in the substrates used for the production of energy. Indeed, early perfusion experiments demonstrated that for diabetic rodents, cardiac metabolism was characterised by increased utilisation of fatty acids and triacylglycerol [5], despite the high levels of plasma glucose. Similar observations have also been noted for skeletal muscle [6], highlighting the fundamental role demonstrated by insulin in both glucose and, indirectly, fatty acid metabolism.

Current therapies for diabetes are characterised by interventions to normalise the utilisation of energy substrates by tissues. For example, the anti-hyperglycaemic drug metformin triggers signals of relative energy deprivation, stimulating glucose utilisation for energy production [7]. Thiazolidinediones stimulate the uptake of glucose via adipose tissue, facilitating lipogenesis for enhanced deposition of glucose [8]. New therapeutics, demonstrating more modest side effects, to manipulate the control of metabolism may offer new mechanisms to diminish the negative effects of hyperglycaemia. These may include direct inhibition of lipid oxidation, which may help to normalise blood glucose.

Tissue carnitine levels may represent one such potential target, as carnitine is an obligate cofactor in fatty acid oxidation. Carnitine supplementation for diabetic patients was previously noted to decrease plasma glucose and lipid levels [9]. However, controversy remains over the potential for carnitine supplementation to decrease blood glucose, with both decreased plasma glucose [10] and no change [11] previously observed. We have previously demonstrated that manipulation of tissue carnitine levels through carnitine supplementation led to increased cellular glucose utilisation in the myocardium, manifesting as increased flux through pyruvate dehydrogenase [12], suggesting that carnitine may be protective for periods of ischaemia. Interestingly, limited data supports the use of carnitine synthesis as a target for manipulation. Mildronate, an inhibitor of ɣ-butyrobetaine hydroxylase (an intermediate in carnitine synthesis), was previously associated with decreased hyperglycaemia in diabetes and plasma triacylglycerol [13], suggesting that blunting lipid oxidation may facilitate enhanced glucose consumption. Moreover, direct inhibition of carnitine synthesis with mildronate (meldonium) normalised blood glucose levels for a rodent model of Type II diabetes [14]. Following development of streptozotocin diabetes (STZ diabetes), mildronate treatment led to decreased blood glucose, coupled with increased glucose transporter (GLUT1) expression [15], suggesting increased glucose utilisation following the blunting of fatty acid oxidation. In addition, direct inhibition of the pathway for carnitine synthesis with mildronate also increased flux through pyruvate dehydrogenase in perfused hearts from both untreated and diabetic rats, implying that alteration of mitochondrial fatty acid metabolism increased the utilisation of glucose [16]. Furthermore, STZ diabetes was also associated with decreased rates of protein synthesis [17] and diversion of branched-chain amino acids to central energy metabolism, suggesting a role in anaplerosis, supporting TCA cycle metabolism [18]. We postulate that blunting of fatty acid oxidation through decreased synthesis of carnitine may increase utilisation of glucose and help to normalise blood glucose with direct and distinct effects on different tissues. This will reflect individual tissue dependence upon both glucose and fatty acids for metabolic fuel. Indeed, the brain demonstrated modest potential to oxidise fatty acids, primarily relying on glucose oxidation for energy production [19], whereas the liver exhibited extensive fatty acid beta-oxidation potential. Mildronate was previously noted to blunt the cognitive decline associated with both ageing [20] and diabetes [21], suggesting a more prominent role for lipid oxidation in brain tissue. More extensive metabolic perturbations were recorded for the liver [22]. What is unclear is whether these changes reflect altered central energy metabolism as a consequence of diabetes and further perturbed by mildronate. Furthermore, accumulation of acyl-carnitines through direct inhibition of beta-oxidation may further contribute to insulin resistance [23]. No previous study has elucidated the effect of mildronate on the tissue metabolome for multiple tissues, quantifying metabolite changes to multiple pathways. Hence, using a metabolomics-based approach, exploiting multiple liquid chromatography platforms, we explored global metabolic changes associated with mildronate treatment in order to explore plasma and tissue (liver and brain) metabolites, estimating carnitine and acyl-carnitine levels and characterising changes to glucose metabolic pathways. The brain represents an obligate consumer of glucose [24], whereas the liver utilises both glucose and fatty acids for metabolic processes. We postulate that the brain metabolome will be less disrupted than the liver through a decreased reliance on carnitine-mediated fatty acid oxidation. In addition, the inhibition of carnitine synthesis may lead to accumulation of CoA esters of fatty acids, depleting tissue concentrations of coenzyme A with possible further effects on intermediary metabolism [25].

## 2. Materials and Methods

### 2.1. Materials

Mildronate was purchased from Grindex (Riga, Latvia). Wistar rats were obtained from Harlan Laboratories (Kent, UK). Streptozotocin (STZ) was purchased from Sigma-Aldrich (St. Louis, MO, USA).

### 2.2. Methods

#### 2.2.1. Animal Experimentation Ethical Approvals

All procedures (i) had local approval and (ii) conformed to the guidelines from Directive 2010/63/EU of the European Parliament on the protection of animals used for scientific purposes or the NIH Guide for the Care and Use of Laboratory Animals. Animal studies were conducted in accordance with the UK Animals (Scientific Procedures) Act (1986) (https://www.legislation.gov.uk/ukpga/1986/14/contents, accessed on 12 December 2025), PPL Number 30/3322, and local ethical guidelines (Medical Research Council Responsibility in the Use of Animals for Medical Research, July 1993).

Thirty-six healthy male Wistar rats (~200 g: 6 weeks old) were randomly divided into four treatment groups. Animals were housed in groups of *n* = 6 with ad libitum access to food and water. All animals were fasted overnight (food removed for 12–15 h) and then either made diabetic with one intraperitoneal (i.p.) injection of streptozotocin (STZ, 55 mg/kg) or kept as controls via an injection of citrate buffer. Diabetes was confirmed by ensuring glucose levels higher than 110 mmol/L in urine samples using reagent test strips (Dirui, Jilin, China) for two consecutive days following STZ injection and by establishing blood glucose levels higher than 8.3 mmol/L (Accu-Chek Aviva, Roche, Basel, Switzerland) at 6 d after STZ injection. Two weeks after STZ/citrate buffer injection, all animals were initiated on daily morning i.p. treatment with either saline or meldonium (100 mg/kg/day). Mildronate treatment was continued for a further 14 days.

#### 2.2.2. Plasma Metabolites

Post-mortem, anaesthesia was induced in isoflurane (3% in oxygen), and following laparotomy, terminal blood samples were drawn from the inferior vena cava into heparinised syringes and plasma was collected following centrifugation. Plasma was collected for all groups and used to measure glucose (Thermo-Electron glucose oxidase kit) and insulin (ultra-sensitive Rat insulin ELISA kit, Mercodia, Uppsala, Sweden).

#### 2.2.3. Tissue Metabolites

Tissue samples were excised, immediately placed into liquid nitrogen and stored at −80 °C until further analysis. Tissue was ground in liquid nitrogen, and tissue (~100 mg) was extracted in methanol/water (80:20). Following homogenisation, supernatants were recovered and DNA concentration estimated using a nanodrop spectrophotometer (Thermo Fisher, UK). The resulting samples were diluted to yield equal DNA concentration in all samples.

#### 2.2.4. Ion-Exchange Chromatography

In brief, each sample was analysed using up to three separate liquid chromatography with tandem mass spectrometry (LC-MS/MS) methods using two different LC systems (Thermo Scientific ICS-5000+ ion chromatography system and a Thermo Ultimate 3000, Waltham, MA, USA). Each was coupled directly to a Q-Exactive HF Hybrid Quadrupole-Orbitrap mass spectrometer with a HESI II electrospray ionisation source (Thermo Scientific, San Jose, CA, USA). The IC-MS/MS was performed using an ICS-5000+ HPLC system incorporating an electrolytic anion generator (KOH) which was programmed to produce an OH gradient prior to MS analysis (Thermo Scientific Dionex AERS 500, Sunnyvale, CA, USA) [26].

Plasma samples were filtered through molecular weight cut-off filters (10 kd) to remove proteins The infranatant was recovered and divided, with one aliquot evaporated to dryness under reduced pressure. Sample residue was then resuspended in acetonitrile/water (95%:5%) (HILIC method). Authenticated standards for selected acyl-carnitines (up to 1.0 μg/mL) were prepared using an identical method. The remaining aliquot was stored for direct analysis of metabolites.

#### 2.2.5. C18 Reverse-Phase Negative Ion Mass Spectrometry

Chromatographic separation was performed using a Dionex Ultimate 3000 UHPLC system (Dionex, Sunnyvale, CA, USA) coupled to a Q-Exactive HF hybrid quadrupole-Orbitrap mass spectrometer (Thermo Scientific, San Jose, CA, USA). A CORTECS T3 C18 column (2.1 × 100 mm^2^, 1.6 μm; Waters, Milford, MA, USA) at 40 °C was used with mobile phase A—water with 0.1% (*v*/*v*) aqueous formic acid—and mobile phase B—methanol with 0.1% (*v*/*v*) aqueous formic acid. The linear gradient used was as follows: 0 min, 5% B; 4.0 min, 50% B; 12.0 min 99.9% B; 14.0 min, 99% B; and 15.1 min, 5% B. The flow rate was 0.3 mL/min, and the total run time was 18 min. The mass spectrometer was equipped with a HESI II probe in negative ion mode with source parameters set as follows: sheath gas flow rate, 25; auxiliary gas flow rate, 8; sweep gas flow rate, 0; spray voltage, 3.5 kV; capillary temperature, 300 °C; S-lens RF level, 70; and heater temperature, 300 °C. MS scan parameters were set as follows: microscans, 2; resolution, 7 × 104; AGC target, 5 × 106 ions; maximum IT, 120 ms; and scan range, 60–900 *m*/*z*. MS/MS scan parameters were set as follows: microscans, 2; resolution, 1.75 × 104; AGC target, 1 × 105 ions; maximum IT, 80 ms; loop count, 10; MSX count, 1; isolation window, 2.0 *m*/*z*; collision energy, 35; minimum AGC target, 5 × 103 ions; charge exclusion, 3–8, >8; and dynamic exclusion, 20.0 s [27].

#### 2.2.6. C18 Reversed Phase (Derivatised)

The LC-MS method used a sample derivatisation protocol to label 1° and 2° amines followed by analysis based on a modified version of the Waters AccQ-Tag method [28]. C18 reversed-phase analysis of derivatised samples was also performed using the Thermo Utimate 3000 UHPLC system coupled directly to a Q-Exactive HF Hybrid Quadrupole-Orbitrap mass spectrometer. A 5 μL partial-loop injection was used for all analyses with a pre- and post-injection wash programme. A Waters AccQ-Tag column (2.1 × 100 mm) was used with a flow rate of 0.5 mL/min. The total run time was 9.5 min. Mobile phase A and B comprised commercially available AccQ-Tag reagents prepared as recommended by Waters (Waters PLC, Elstree, UK). The gradient elution program was modified from the published AccQ-Tag method as follows: 0 mins, 0.1%B; 0.54 min, 9.1%B; 5.74 min, 21.2%B; 7.74 mins, 59.6%B; 8.04 min, 90%B; 8.05 min, 90%B; 8.64 min, 0%B; and 9.5 min, 0.1%B. The column temperature was kept at 40 °C throughout the experiment. Mass spectrometry analysis was performed in positive ion mode separately using a scan range from *m*/*z* 70 to 1050 and resolution set to 70,000. The tune file source parameters were set as follows: sheath gas flow, 60 mL/min; Aux gas flow, 20 mL/min; spray voltage, 3.6 v; capillary temperature, 320 °C; S-lens RF value, 70; and heater temperature, 350 °C. The full MS settings were as follows: the AGC target was 3e6 ions, and the Max IT value was 200 ms. Full scan data were acquired in continuum mode.

#### 2.2.7. Hydrophobic-Interaction Liquid Chromatography (HILIC)–MS

Chromatographic separations were performed using a Dionex Ultimate 3000 UHPLC system (Dionex, Sunnyvale, CA, USA) coupled to a Q-Exactive HF hybrid quadrupole-Orbitrap mass spectrometer (Thermo Scientific, San Jose, CA, USA). A BEH Amide column (2.1 × 100 mm, 1.7 μm; Waters, Milford, MA, USA) at 25 °C was used with mobile phase A—95% acetonitrile (*v*/*v*) aqueous solution containing 10 mM ammonium acetate—and mobile phase B—50% acetonitrile (*v*/*v*) aqueous solution containing 10 mM ammonium acetate. The linear gradient used was as follows: 0 min, 1% B; 1.0 min, 1% B; 6.0 min 45% B; 10.0 min, 95% B; 12.0 min, 99% B; 12.1 min, 1% B; and 15.0 min, 1% B. The flow rate was 0.4 mL/min, and the total run time was 15 min. The mass spectrometer was equipped with a HESI II probe operating in negative and positive ion modes with source parameters set as follows: sheath gas flow rate, 25; auxiliary gas flow rate, 8; sweep gas flow rate, 0; spray voltage, ±3.5 kV; capillary temperature, 300 °C; S-lens RF level, 55; and heater temperature, 300 °C. MS scan parameters were set as follows: microscans, 2; resolution, 7 × 104; AGC target, 5 × 106 ions; maximum IT, 120 ms; and scan range, 60–900 *m*/*z*. MS/MS scan parameters were set as follows: microscans, 2; resolution, 1.75 × 104; AGC target, 1 × 105 ions; maximum IT, 80 ms; loop count, 10; MSX count, 1; isolation window, 2.0 *m*/*z*; collision energy, 35; minimum AGC target, 5 × 103 ions; charge exclusion, 3–8, >8; and dynamic exclusion, 20.0 s. Putative compounds were identified with reference to authenticated standards for selected acyl-carnitines using retention time, accurate mass and fragmentation pattern to identify individual compounds [26]. Concentrations were calculated with reference to specific standard curves.

### 2.3. Data Processing

Ion species were identified with reference to an ‘in-house’ database created from authenticated standards. Briefly, pure compounds were purchased from chemical suppliers (e.g., Sigma-Aldrich, Gillingham, UK; Tocris, Bristol, UK; Tokyo Chemicals industry, Oxford, UK). These standards were then diluted in an appropriate solvent (80% methanol) and separated chromatographically by different methods. Each compound was then examined using a QExactive Mass Spectrometer (Thermo, Altringham, Cheshire, UK). Each authenticated standard was identified by collection of discrete data: this included chromatographic retention time, accurate mass (5 decimal places) and compound fragmentation, hence allowing the identification of different structural isomers with reference to differing fragmentation and retention characteristics.

Raw data files were processed using ProgenesisQI (Waters, Elstree, UK). This process included alignment of retention times, peak picking by identification of the presence of natural abundance isotope peaks, characterisation of multiple adduct forms and identification of metabolites using our in-house database. Retention times, accurate mass values, relative isotope abundances and fragmentation patterns were compared between authentic standards and the samples measured. Identifications were accepted only when the following criteria were met: <5 ppm differences between measured and theoretical mass (based on chemical formula), <30 s differences between authenticated standard and analyte retention times, and isotope peak abundance measurements for analytes that were >90% matched to the theoretical value generated from the chemical formula. Where measured, fragmentation patterns were matched to at least the base peak and two additional peak matches in the MS/MS spectrum to within 12 ppm.

### 2.4. Statistical Analysis

Analysis of raw ion feature data was undertaken using MetaboAnalyst 6.0. Data was filtered by interquartile range, normalised and scaled (mean-centring/SD) [28]. Data normalisation was undertaken using ‘autoscaling’ [29]. Metabolomic results between the treatments and the control were analysed using univariate statistical analysis determining fold change and *t*-tests between experimental groups for compound features and combined in volcano plots (accounting for FDR-adjusted *p*-values). PCA [30] and PLS-DA were also used to analyse the patterns in metabolomic profiles among treatments. Data is presented as the mean ± SD. Statistical significance was assigned at *p* ≤ 0.05. Two-factor ANOVA analysis was undertaken using MetaboAnalyst 6.0 [31,32] to determine the interaction between STZ diabetes and mildronate.

## 3. Results

### 3.1. Animal Husbandry

Following STZ administration, rats gained weight more slowly than untreated controls and showed hyperphagia and polyuria). Routine blood tests indicated hyperglycaemia following STZ treatment that was confirmed post-mortem, with STZ-diabetic rats indicating a 50% increase in plasma glucose (*p* < 0.001; Table 1) coupled with an 80% decrease in plasma insulin concentrations (*p* < 0.001; Table 1). Supplementation of STZ-diabetic rats with mildronate restored blood glucose to control levels (NS compared to untreated; Table 1). Multiple mass spectrometry workflows were exploited to identify ion species in different tissues (Table S1).

Mildronate administration increased plasma mildronate concentration to 40 ± 0.14 µg/mL, with a plasma concentration in STZ-diabetic rats of 32 ± 2.2 µg/mL (NS; Table 1). Tissue concentrations of mildronate were estimated with reference to an authenticated standard, with liver concentration being 213 ± 37 µg/gm and brain concentration estimated as 281 ± 61 µg/gm (Table 1). STZ diabetes had no effect on tissue mildronate concentrations (NS; Table 1). Development of STZ diabetes led to a significant decrease in plasma carnitine (*p* = 0.026; Table 1), which was further decreased following supplementation with mildronate (*p* = 0.006). STZ diabetes also decreased plasma acyl-carnitines with a 50% decrease in C6-acyl-carnitine (*p* < 0.001), C3-acyl-carnitine (*p* < 0.001) and C14-acyl-carnitine (*p* < 0.001; Table 1). Mildronate exposure led to a further decrease in acyl-carnitine abundance, with a 75% reduction for C6-acyl-carnitine (*p* = 0.002), C3-acyl-carnitine (*p* = 0.0055) and C14-acyl-carnitine (*p* = 0.022; Table 1) compared with untreated controls. For C18:0- and C18:1-acyl-carnitines, mildronate exposure decreased abundance by two-thirds compared with control plasma (Table 1).

Estimation of hepatic acyl-carnitine abundance demonstrated that STZ diabetes increased coenzyme A (*p* < 0.001; Table 2). However, mildronate had no effect on coenzyme A levels. For both carnitine and butyryl-carnitine, mildronate treatment significantly decreased abundance (*p* < 0.001; Table 2), with no effect noted following STZ-diabetes development.

STZ-diabetes development led to a decrease in abundance of methylmalonyl-carnitine (*p* < 0.05) and was contrasted by a 2-fold increase in octanoyl-carnitine (*p* < 0.05; Table 3) for brain tissue. In addition, mildronate treatment led to a significant decrease in abundance of carnitine, hexanoyl-carnitine and octanoyl-carnitine (*p* < 0.001; Table 3) with more modest decreases in stearoyl-carnitine and 3-hydroxybutyryl-carnitine (*p* < 0.01; Table 3). Mildronate treatment was associated with decreased acetyl-carnitine and tetradecanoyl-carnitine (*p* < 0.05).

### 3.2. Metabolic Pathway Enrichment

**Figure 1:**
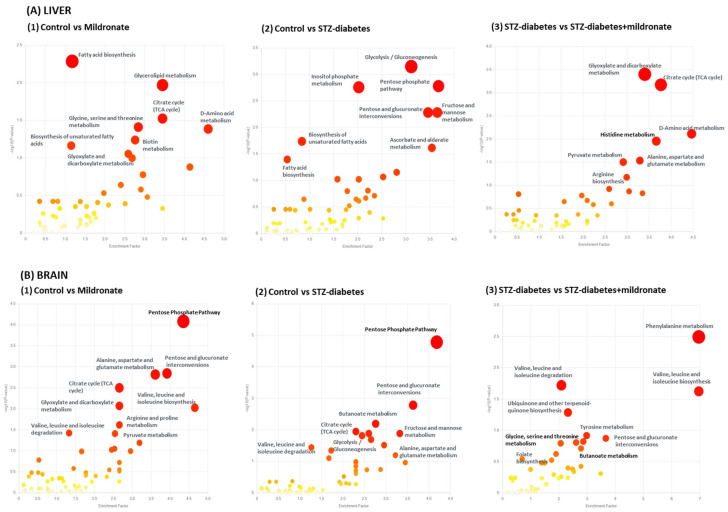
Effects of STZ diabetes and mildronate treatment on the metabolic pathway enrichment for individual metabolic pathways. Pathways were identified from individual ion species detected by ion-exchange mass spectrometry. Intensity of colouration is indicative of decreasing −logP value as detailed by the Y-axis. Size of the circle is indicative of the proportion of ion species identified that were assocaited with individual pathways. For binary comparisons [untreated vs. mildronate (**1**); untreated vs. STZ diabetes (**2**); STZ diabetes vs. STZ diabetes + mildronate (**3**)] for liver (**A**) and brain tissue (**B**), fold change and unpaired *t*-test calculations were undertaken and identification of ion species was determined using species charge and monoisotopic mass with reference to Mummichog database. Metabolic pathway enrichment was estimated using Metaboanalyst 6.0. Data represents group sizes of *n* = 6 for all groups. For further details, see Section 2.2.

Principal component analysis and Partial Least Squares discrimination analysis was undertaken on all ion features for plasma, liver and brain (Table S2) and summarised (Figure S1). To identify putative metabolic pathway perturbations, metabolite enrichment analysis was undertaken exploiting binary comparisons for ion features coupled with fold change and statistical analysis [33]. These ion features were matched to identified compounds in the Mummichog database based on *m*/*z* value [34] and were matched with individual pathways to determine putative targets [35] (Figure 1). Binary comparisons were undertaken to determine the effect of mildronate (1), the effect of STZ diabetes (2) and the impact of mildronate on STZ diabetes (3). Analysis of liver tissue (Figure 1A) demonstrated that mildronate treatment affected central energy metabolism with altered TCA cycle metabolites (*p* = 0.03), amino acid metabolism (*p* = 0.041) and glycine, threonine and serine metabolism (*p* = 0.039). Development of STZ diabetes (Figure 1A(2)) significantly affected metabolic pathways including glycolysis/gluconeogenesis (*p* = 7.09 ×10^−4^), the pentose phosphate pathway (*p* = 0.002) and pentose and glucuronate metabolism (*p* = 0.005). For STZ-diabetic rats, supplementation with mildronate led to altered TCA cycle metabolism (*p* = 7.61 × 10^−4^), amino acid metabolism (*p* = 0.008) and pyruvate metabolism (*p* = 0.031). Metabolites were identified with reference to an ‘in house’ database of authenticated standard compounds and 2-Factor ANOVA analysis undertaken for identified compounds from plasma (Table S3), liver (Table S4) and brain (Table S5).

Analysis of brain metabolites indicated that mildronate (Figure 1B(1)) disrupted the pentose phosphate pathway (*p* = 8.26 × 10^−5^), pentose and glucuronate interconversion (*p* = 0.001) and alanine glutamate and aspartate metabolism (*p* = 0.002). Following development of diabetes with STZ (Figure 1B(2)), the pentose phosphate pathway (*p* = 1.65 × 10^−5^) was affected by diabetes, with pentose and glucuronate interconversion also affected (*p* = 0.002). For STZ-diabetic rats, mildronate (Figure 1B(3)) affected amino acid metabolic pathways, with phenylalanine metabolism (*p* = 0.003), valine, leucine and isoleucine degradation (*p* = 0.019) and valine, leucine and isoleucine biosynthesis (*p* = 0.024) being putative metabolic targets.

### 3.3. TCA Cycle

**Figure 2:**
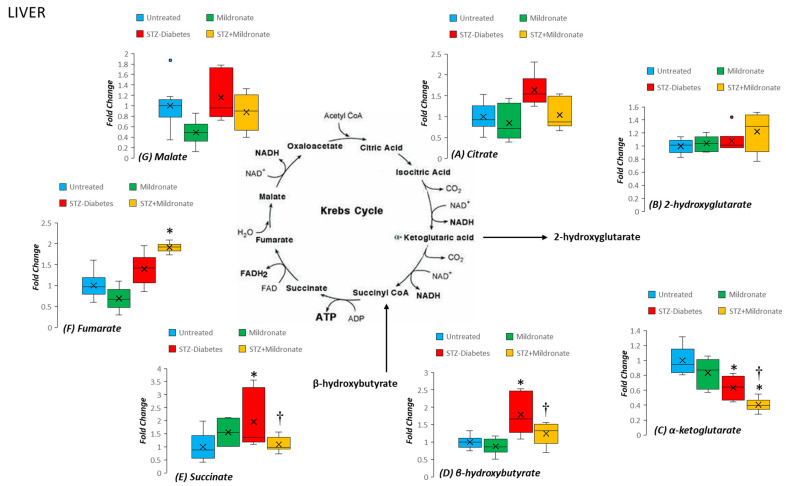
Effect of diabetes and mildronate treatment on metabolite abundance for TCA cycle in rat liver. Metabolites represented as (**A**) Citrate, (**B**) 2-hydroxyglutarate, (**C**) α-ketoglutarate, (**D**) β-hydroxybutyrate, (**E**) succinate, (**F**) fumarate and (**G**) malate. Coloured datapoints represent outlier data for each experimental group. Data represents fold change calculated for identified ion features with reference to authenticated standards. Identification was undertaken with reference to accurate mass, retention time and fragmentation pattern (for further details, see Section 2.2). Data is represented in box and whisker plots (*n* = 12 for untreated; *n* = 6 for STZ diabetes and mildronate). Statistical analysis was undertaken by ANOVA, with data presenting * *p* < 0.05 for the effect of diabetes and † *p* < 0.05 for the effect of mildronate.

Hepatic α-ketoglutarate abundance was halved by STZ diabetes (*p* < 0.05; Figure 2), and supplementation with mildronate further halved abundance (*p* < 0.05; Figure 2). STZ diabetes also increased hepatic β-hydroxybutyrate 50% (*p* < 0.05; Figure 2) compared with the untreated group; however, supplementation with mildronate restored β-hydroxybutyrate levels to untreated levels (*p* < 0.05; Figure 2). As a consequence, STZ diabetes doubled hepatic succinate levels (*p* < 0.05; Figure 2), with mildronate treatment returning tissue succinate abundance to untreated levels (NS; Figure 2). In addition, supplementation with mildronate further increased fumarate by one-third (*p* < 0.05; Figure 2) compared with STZ diabetes alone.

**Figure 3:**
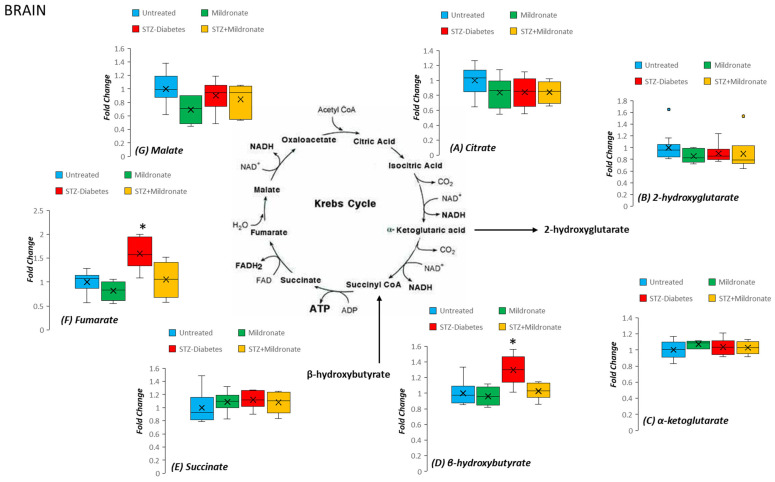
Effect of diabetes and mildronate treatment on metabolite abundance for TCA cycle in rat brain. Metabolites represented as (**A**) Citrate, (**B**) 2-hydroxyglutarate, (**C**) α-ketoglutarate, (**D**) β-hydroxybutyrate, (**E**) succinate, (**F**) fumarate and (**G**) malate. Coloured datapoints represent outlier data for each experimental group. Data represents fold change calculated for identified ion features with reference to authenticated standards. Identification was undertaken with reference to accurate mass, retention time and fragmentation pattern (for further details, see Section 2.2). Data is represented in box and whisker plots (*n* = 12 for untreated; *n* = 6 for STZ diabetes and mildronate). Statistical analysis was undertaken by ANOVA, with data presenting * *p* < 0.05 for the effect of diabetes.

For brain tissue, STZ diabetes led to a modest 20% increase in tissue β-hydroxybutyrate levels (*p* < 0.05; Figure 3), which were restored to untreated levels following supplementation with mildronate (NS; Figure 3). In addition, STZ diabetes increased brain fumarate abundance by 50% compared with untreated tissue (*p* < 0.05; Figure 3).

### 3.4. Amino Acid Abundance

**Figure 4:**
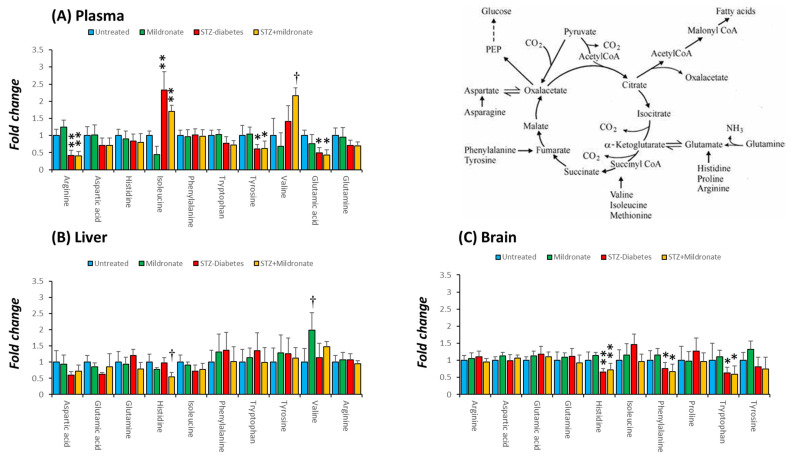
Effect of diabetes and mildronate treatment on metabolite abundance for amino acids supporting anaplerosis in rat plasma (**A**), liver (**B**) and brain (**C**). Data represents fold change calculated for identified ion features with reference to authenticated standards. Identification was undertaken with reference to accurate mass and retention time using derivatised C18 reverse-phase chromatography–mass spectrometry (for further details, see Section 2.2). Data is represented in box and whisker plots (*n* = 12 for untreated; *n* = 6 for STZ diabetes and mildronate). Statistical analysis was undertaken by ANOVA, with data presenting * *p* < 0.05 and ** *p* < 0.01 for the effect of diabetes, and † *p* < 0.05 for the effect of mildronate.

Amino acids represent a substrate for anaplerosis to augment tissue abundance of TCA cycle intermediates. Following development of STZ diabetes, plasma abundance of arginine was halved (*p* < 0.01; Figure 4A). Similar observations were recorded for plasma tyrosine and glutamate abundances following STZ diabetes (*p* < 0.05 for both; Figure 4A). Isoleucine levels were increased 2.5-fold by STZ diabetes (*p* < 0.01; Figure 4A), and this effect was preserved following supplementation with mildronate (NS; Figure 4A). Mildronate treatment of STZ rats led to a 50% increase in the plasma abundance of valine compared with STZ treatment alone (*p* < 0.05; Figure 4A).

For hepatic tissue, supplementation of STZ rats with mildronate led to a halving of liver histidine levels compared with STZ alone (*p* < 0.05; Figure 4B). In addition, mildronate treatment alone increased tissue valine abundance by 100% compared with the control (*p* < 0.01; Figure 4B).

For brain tissue, STZ diabetes halved abundance of tissue histidine (*p* < 0.01; Figure 4C), an effect that was preserved following supplementation with mildronate (*p* < 0.01 compared to control; Figure 4C). In addition, STZ diabetes decreased brain phenylalanine levels by 30% (*p* < 0.05; Figure 4C), and this was maintained following supplementation with mildronate (NS; Figure 4C). Brain tryptophan abundance was also halved following development of STZ diabetes (*p* < 0.05; Figure 4C), and this effect was sustained following supplementation with mildronate (*p* < 0.05 compared with control; Figure 4C).

### 3.5. Oxidative Pentose Phosphate Pathway

**Figure 5:**
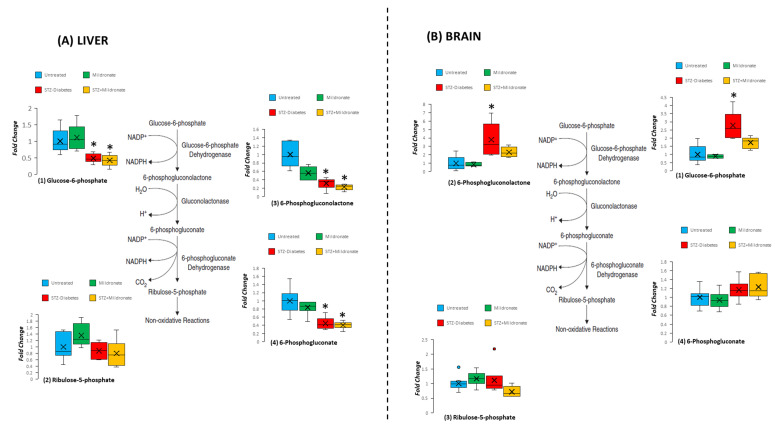
Effect of diabetes and mildronate treatment on metabolite abundance for pentose phosphate pathway in rat liver (**A**) and brain (**B**). Data represents fold change calculated for identified ion features with reference to authenticated standards. Identification was undertaken with reference to accurate mass, retention time and fragmentation pattern (for further details, see Section 2.2). Data is represented in box and whisker plots (*n* = 12 for untreated; *n* = 6 for STZ diabetes and mildronate). Statistical analysis was undertaken by ANOVA, with data presenting * *p* < 0.05 for the effect of diabetes.

The oxidative component of the pentose phosphate pathway is responsible for the reduction of NADP to facilitate biosynthetic reactions. For hepatic tissue, STZ diabetes halved tissue abundance of glucose-6-phosphate (*p* < 0.05; Figure 5A). By contrast, STZ-diabetes development decreased 6-phosphogluconate levels to one-third of untreated levels (*p* < 0.05; Figure 5A), and this was maintained following supplementation with mildronate (*p* < 0.05; Figure 5A).

For brain tissue, STZ diabetes increased glucose-6-phosphate 2.5-fold compared with untreated rats (*p* < 0.05; Figure 5B), and this was restored to untreated levels following supplementation with mildronate (NS; Figure 5B). These changes were preserved for 6-phosphogluconolactone, with STZ diabetes yielding a 3-fold increase in 6-phosphogluconolactone abundance (*p* < 0.05; Figure 5B), which was restored to untreated levels following mildronate supplementation (NS; Figure 5B).

### 3.6. Sorbitol Accumulation

**Figure 6:**
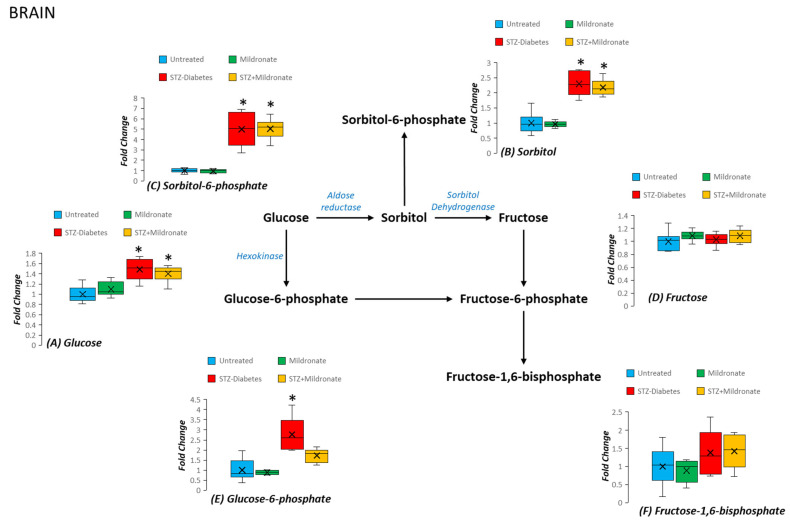
Effect of diabetes and mildronate treatment on metabolite abundance for sorbitol metabolites in rat brain. Metabolites represented as (**A**) glucose, (**B**) sorbitol, (**C**) sorbitol-6-phosphate, (**D**) fructose, (**E**) glucose-6-phosphate and (**F**) fructose-1,6-bisphosphate. Data represents fold change calculated for identified ion features with reference to authenticated standards. Identification was undertaken with reference to accurate mass, retention time and fragmentation pattern (for further details, see Methods). Data is represented in box and whisker plots (*n* = 12 for untreated; *n* = 6 for STZ diabetes and mildronate). Statistical analysis was undertaken by ANOVA, with data presenting * *p* < 0.05 for the effect of diabetes.

Sorbitol represents a component of the polyol pathway and is associated with STZ-diabetes development. We note that STZ diabetes was associated with a 40% increase in brain glucose (*p* < 0.05; Figure 6A). Tissue abundance of sorbitol was doubled following STZ-diabetes development (*p* < 0.05; Figure 6B), which was not affected by the supplementation with mildronate (NS compared with STZ diabetes; Figure 6B). In addition, STZ diabetes was associated with a 5-fold increase in sorbitol-6-phosphate (*p* < 0.05; Figure 6C) that was also sustained following treatment with mildronate (NS compared with STZ; Figure 6C). Tissue abundance of glucose-6-phosphate was increased 2.5-fold for the brain following development of STZ diabetes (*p* < 0.05; Figure 6E). A strong correlation was also noted between brain glucose and sorbitol abundance (r^2^ = 0.734; Figure S2A) and sorbitol and sorbitol-6-phosphate (r^2^ = 0.814; Figure S2C).

### 3.7. Glycolysis

**Figure 7:**
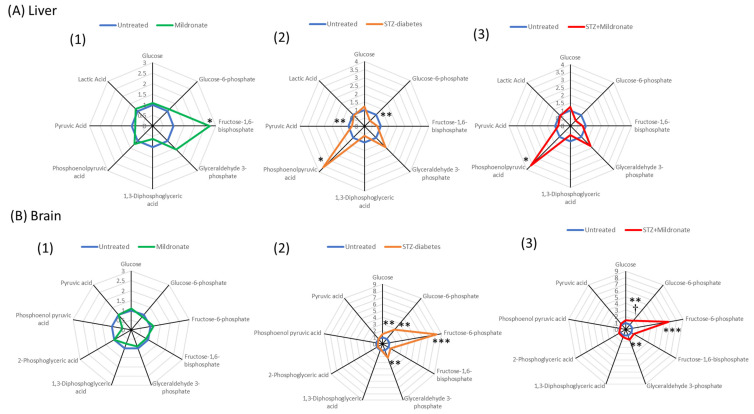
Effect of diabetes and mildronate treatment on metabolite abundance for glycolytic pathway metabolites in rat liver (**A**) and brain (**B**). Data represents binary comparisons [untreated vs. mildronate (**1**); untreated vs. STZ diabetes (**2**); STZ diabetes vs. STZ diabetes + mildronate (**3**)] for fold change calculated for identified ion features with reference to authenticated standards. Identification was undertaken with reference to accurate mass, retention time and fragmentation pattern (for further details, see Section 2.2). Data is represented in target plots (*n* = 12 for untreated; *n* = 6 for STZ diabetes and mildronate). Statistical analysis was undertaken by ANOVA, with data presenting * *p* < 0.05, ** *p* < 0.01 and *** *p* < 0.001 for the effect of diabetes, and † *p* < 0.05 for the effect of mildronate.

Glycolysis facilitates the synthesis of pyruvate to support the TCA cycle and also produces reduced cofactors for the synthesis of ATP. For hepatic tissue, mildronate increased liver fructose-1,6-bisphosphate levels 2.5-fold (*p* < 0.05; Figure 7A(1)). Following development of STZ diabetes, liver glucose-6-phosphate halved compared with the untreated control (*p* < 0.01; Figure 7A(2)), with a 20% decrease in pyruvate abundance (*p* < 0.01; Figure 7A(2)). STZ-diabetes development also led to a 3.5-fold increase in hepatic phosphoenol pyruvate abundance (*p* < 0.05; Figure 7A(2)) compared with the untreated group.

For brain tissue, after development of STZ diabetes, glyceraldehyde-3-phosphate showed a 2-fold increase in abundance (*p* < 0.01; Figure 7B(2)), and this was accompanied by a 1.5-fold increase in brain glucose abundance (*p* < 0.01; Figure 7B(2)), a 2.5-fold increase in glucose-6-phosphate (*p* < 0.01; Figure 7B(2)) and an 8-fold increase in brain fructose-6-phosphate (*p* < 0.001; Figure 7B(2)). The effect of mildronate in brain tissue of STZ-treated rats was characterised by a halving of brain glucose-6-phosphate compared with STZ alone (*p* < 0.05; Figure 7B(3)).

## 4. Discussion

Diabetes is characterised by elevated plasma glucose and diminished efficacy of insulin. We demonstrate that manipulation of metabolism with mildronate to increase the utilisation of glucose showed the potential to normalise blood glucose concentrations, with mildronate decreasing blood glucose concentrations without altering the plasma insulin levels, confirming previous observations [36]. Given the potency of mildronate to disrupt the endogenous synthesis of carnitine, we also demonstrated that the manipulation of tissue fatty acid metabolism increased reliance on glucose consumption for the provision of ATP. Furthermore, we note that metabolite perturbation was greater for tissue with reliance on fatty acid oxidation for ATP provision, e.g., liver. Whilst liver tissue showed significant disruption of metabolism as a consequence of mildronate exposure, the brain, an obligate consumer of glucose, appeared relatively resistant to disruption of metabolism. Of note, we record that mildronate could be identified in both liver and brain tissue, suggesting that metabolism in these respective tissues was a likely target for disrupted CPT1-mediated metabolism. Hence, the limited disruption of metabolism for the brain reflected the limited reliance on fatty acid oxidation. Early experiments highlighted that the brain’s concentration of carnitine [13] is only 20% of the concentration in the liver [15], implying a decreased reliance on lipids for energy production in the brain. In support of this, we also observe that tissue carnitine levels were further decreased by mildronate exposure. Previous experiments highlight the potential for mildronate uptake facilitated by the carnitine transporter SLC22A5 given their structural similarity, and this has been identified in both the blood–brain barrier [37] and liver [38]. We demonstrate the potency of mildronate to normalise blood glucose against a background of STZ diabetes, suggesting that mildronate promoted the utilisation of glucose as substrate rather than affecting the plasma burden of insulin or restoring pancreatic beta-cell function, thus supporting our hypothesis. This recapitulates the observations previously noted for models of Type II diabetes and for Zucker Obese Fatty rats (ZDF) [14]. Indeed, mildronate may also affect protein expression, increasing the abundance of glucose transporters following diabetes to facilitate increased glucose uptake [15,39]. Interestingly, tissue-specific analysis of insulin receptor expression following mildronate exposure demonstrated that mildronate augmented expression of insulin receptor in the heart [39]. This suggests the potential to increase insulin-stimulated glucose uptake, with particular importance for models of non-insulin-dependent diabetes and obesity. Mildronate treatment decreased plasma, liver and brain carnitine concentrations for both untreated and STZ-diabetic rats, supporting previous observations [40]. Moreover, given the obligate nature of carnitine as a cofactor for fatty acid metabolism, we predicted a decrease in fatty acid oxidation potential, promoting the utilisation of glucose.

### 4.1. Plasma Acyl-Carnitine Levels

We noted that plasma acyl-carnitine levels were also manipulated by both diabetes and mildronate treatment. However, this was observed for only certain acyl-carnitines. Plasma carnitine concentrations were depleted following development of STZ diabetes, and this was also true for C3-acyl-carnitine in addition to C6- and C14-acyl-carnitines. In part, this may reflect the depletion of carnitine by diabetes and may also result from enhanced metabolism coupled with enhanced renal clearance [22]. A significant proportion of propionic acid (C3) may originate from the gut microbiome, and the associated hyperphagia during STZ diabetes may also alter propionate production. Furthermore, given the high rate of fat oxidation in STZ diabetes by oxidative tissues, carnitine depletion may directly influence the utilisation of fatty acids from plasma [41]. Of note was the decrease in plasma C6- and C14-acyl-carnitines. These represent a class of medium-chain-length fatty acids that show potential for transporter-free diffusion across cell membranes. The decreases in abundance promoted by mildronate treatment may reflect decreased beta-oxidation rates increasing the potential for loss from mitochondria coupled with the increased utilisation induced by STZ diabetes. Together, these findings implied that the available pool of these labile fatty acid intermediates is further depleted. We also observed that STZ diabetes resulted in increased metabolite abundance for ketoadipic acid and beta-hydroxybutyrate, which may contribute to the organic acidosis associated with STZ diabetes [42].

Of note was the decrease in plasma carnitine levels as a result of STZ-diabetes development alone. Previous observations indicate that diabetes was, in part, associated with increased renal clearance of acyl-carnitines [43]. Indeed, loss of acyl-carnitines may be indicative of diabetic nephropathy potentially contributing to carnitine insufficiency. Furthermore, STZ diabetes blunted expression of hepatic γ-butyrobetaine hypdroxylase, highlighting decreased de novo carnitine synthesis [44].

Beta-hydroxybutyrate increased as a consequence of STZ diabetes, through increased fatty acid flux into the liver. Interestingly, mildronate had limited effect on plasma beta-hydroxybutyrate levels, advancing the limited effects of mildronate on hepatic ketogenesis, proposing elevated ketone body production in excess of consumption. Elevated beta-hydroxybutyrate abundance noted for the brain following STZ-diabetes development reflected the high plasma concentration facilitating diffusion into the brain, supporting the increased metabolite abundance for the TCA cycle, particularly distal to the succinyl-CoA Oxoacid Transferase step [45], supporting oxidative metabolism, evidenced by increased succinate and fumarate. Of note was the effect of mildronate on 3-hydroxybutyryl-carnitine, blunting its abundance. Given that hydroxybutyryl-carnitine was associated with ketosis [46], this implies the potential for mildronate to depress ketosis.

Early experiments outlined that the brain’s mitochondria may be resistant to the effects of STZ on metabolic function [47]. However, STZ demonstrated the potential to increase the permeability of the blood–brain barrier, leading to altered metabolite abundances, diminishing metabolite gradients between the brain and blood. Tissue free amino acid abundance for the brain was previously noted to be unchanged following development of STZ diabetes [48], supporting the observations that we record. By contrast, examination of brain metabolites for db/db mice as a model for Type II diabetes suggests that citrate and succinate abundances are decreased, coupled with increased lactate, implying a decreased TCA cycle and increased glycolysis contribution to ATP production [49].

### 4.2. Liver

Our experiments represented the first demonstration of mildronate uptake and accumulation in both the brain and liver. We observed a decrease in hepatic ketone body abundance following carnitine depletion with mildronate, suggesting that carnitine depletion diminished rates of ketosis and hence protected tissues from ketoacidosis associated with diabetes (through decreased rates of β-hydroxybutyrate synthesis) [50]. Carnitine depletion was previously reported to increase hepatic expression of mitochondrial fatty acid-oxidising enzymes despite decreased rates of β-oxidation, highlighting the essentiality of carnitine. Early experiments indicated that STZ diabetes was associated with increased hepatic expression of acetoacetate synthetase, supporting the increased ketone body abundance noted for plasma [51].

Elevated hydroxy-3-methylglutarate (HMG) for plasma and the liver may also be a product of elevated ketosis rates in STZ diabetes. Indeed, HMG-CoA synthase represents the controlling enzyme for rates of ketosis [52]. Moreover, STZ diabetes was noted to decrease conversion of HMG-CoA to acetoacetate [53] without changes to HMG-CoA synthesis rates, implying an increase in levels of HMG, which we note for plasma. This increase in HMG may also reflect the hyperphagia associated with STZ diabetes, supporting increased rates of synthesis through increased substrate delivery [54] coupled with decreased HMG-CoA reductase activity, previously observed following STZ diabetes [55]. In addition, we note an increase in hepatic coenzyme A abundance following mildronate exposure, suggesting a blunting of ꞵ-hydroxybutyrate synthesis, yielding increased CoA to support intermediary metabolism. CoA represents an essential cofactor in supporting metabolism. Of further note was the decrease in tissue abundance of hypotaurine in both plasma and the liver following STZ-diabetes development. Hypotaurine represents a breakdown product for CoA [56], implying decreased CoA breakdown, facilitating improved coupling of intermediary metabolism. High tissue quinolinic acid abundance following STZ-diabetes development reflected increased catabolism of tryptophan via the kynurenine pathway, associated with increased hepatic activity of α-amino-β-carboxymuconate-semialdehyde decarboxylase [57].

### 4.3. Anaplerosis from Amino Acids

Anaplerosis represents a mechanism to augment the abundance of TCA cycle intermediates from no-carbohydrate sources, including amino acids. Development of STZ diabetes as well as increasing energy production from fatty acids also demonstrates the potential to deplete TCA cycle intermediates, increasing deposition of amino acids into the TCA via anaplerosis. We document the relative depletion of plasma abundance of arginine, tyrosine and glutamate, potentially in support of anaplerosis, with further tissue-specific declines in the brain for histidine, phenylalanine and tryptophan. Interestingly, we record no change to amino acid abundance for the liver, possibly a consequence of the abundant uptake mechanisms for amino acids in this tissue [58] compared with the transit of the blood–brain barrier necessary for the brain. Of note is the potential imbalance in rates of anaplerosis between the brain and liver. The relatively much higher contribution of fatty acid oxidation for the liver would imply that anaplerosis may make a greater contribution to the liver’s TCA cycle metabolism than the brain’s, as demonstrated by changes to hepatic TCA intermediate abundance, coupled with the relative preservation of abundance in the brain. Interestingly, mildronate increased plasma levels of valine, suggesting that promoting glucose assimilation into TCA cycle intermediates demonstrated a ‘sparing effect’ with regard to valine abundance, highlighting the contribution to anaplerosis made by branched-chain amino acids in STZ diabetes [59]. Of further note was decreased tissue methylmalonyl-CoA for the brain as a consequence of STZ-diabetes development, implying increased utilisation of succinyl-CoA to support the TCA cycle [60].

### 4.4. Pentose Phosphate Pathway

Previous observations indicate that STZ treatment led to increased expression of glucose-6-phosphate dehydrogenase [61], supporting the observation of increased glucose-6-phosphate abundance in the brain that we record, and corroborating the elevated abundance of 6-phosphogluconolactone that we report. Indeed, early experiments demonstrated the direct coupling of the PPP with intermediates of the glycolytic pathway in the brain [62] and demonstrated a neuroprotective effect [63]. Of note was the restoration of G6P when supplemented with mildronate, which may reflect the decrease in blood glucose following mildronate supplementation. In addition, chronic hyperglycaemia was also associated with increased flux through the pentose phosphate pathway, augmenting NADPH synthesis [64]. This may be a direct effect to overcome possible oxidative stress associated with hyperglycaemia [65]. Furthermore, hyperglycaemia was associated with increased sorbitol production, and this was facilitated by increased NADPH utilisation [66], suggesting cofactor utilisation from the pentose phosphate pathway. Interestingly, more recent observations imply that hyperglycaemia was sufficient to increase flux rates through the pentose phosphate pathway, directly augmenting NADPH production [64]. Hyperglycaemia was also characterised by increased oxidative stress [65], and this augmentation of the pentose phosphate pathway may expedite enhanced antioxidant defence.

Following STZ-diabetes development, ketone bodies contributed 45% of total ATP production for the brain, an otherwise obligate glucose-consuming tissue, exploiting high-affinity glucose transporter proteins in the brain. These observations may advocate for ketone bodies to facilitate energy production in the brain despite the decrease in modest levels of brain fatty acid oxidation following carnitine depletion by mildronate.

In contrast to the brain, hepatic tissue demonstrated decreased abundance of glucose-6-phosphate and 6-phosphogluconolactone. Previous experiments demonstrate that STZ treatment led to decreased expression of glucose-6-phosphate dehydrogenase and 6-phosphogluconolactone dehydrogenase [67], supporting our observations. Indeed, this effect may be a direct consequence of the need to preserve flux through the pentose pathway despite the increased rate of glucose uptake [68]. Indeed, GLUT1 expression in the liver was elevated following STZ treatment, coupled with increased GLUT2 expression [69]—augmenting glucose uptake against a background of hyperglycaemia.

### 4.5. Sorbitol Metabolism

Early experiments highlighted increased spinal cord sorbitol concentrations following mild diabetes [70], and we have documented increased brain sorbitol abundance as a consequence of STZ diabetes in common with previously noted experiments, which was not modulated by mildronate exposure despite the potential to decrease plasma glucose concentrations. In addition, hyperglycaemia also led to elevated brain fructose as a result of the polyol pathway [71]. Early experiments highlighted that STZ diabetes had no effect on sorbitol dehydrogenase mRNA or protein expression [72], highlighting that substrate availability may be the predominant factor controlling sorbitol abundance. Indeed, elevated plasma glucose levels correlated with increased sorbitol abundance and restoration of blood glucose to control levels with insulin-depleted tissue sorbitol [73]. Of note was the requirement of NADPH to support aldose reductase activity, facilitating increased brain sorbitol burden [74], implicating coupling with the increased pentose phosphate pathway in brain tissue that we observed.

### 4.6. Glycolysis

Early experiments detailed a decrease in the hepatic flux through glycolysis following the development of STZ diabetes [75]. Our experiments support an increase in metabolites for glycolysis representative of increased glucose abundance, entering hepatocytes from plasma, despite the apparent decrease in glycolytic enzyme expression previously observed. Indeed, STZ diabetes decreased hepatic hexokinase activity, coupled with decreased fructose-1,6-bisphosphatase and glucose-6-phosphatase activity [76]. Together, these observations advance a shift in the balance of metabolism away from glycolysis and glucose oxidation towards hepatic glucose release mediated through gluconeogenesis.

Few studies examined the impact of mildronate on hepatic rates of glycolysis and expression of glycolytic enzymes. We record no direct effect of mildronate on glycolytic metabolite abundance, suggesting that metabolite levels are sufficient to support tissue ATP requirements. Mildronate previously blunted the enhanced expression of PFK mRNA and decreased pyruvate dehydrogenase kinase mRNA expression as a consequence of hypoxia. In addition, decreased lactate dehydrogenase protein abundance was also observed. However, these experiments exploited higher doses of mildronate than those used in the current experiment. For the mouse myocardium, mildronate increased expression of pyruvate dehydrogenase complex and hexokinase II mRNAs [40], further implicating a tissue-specific response for glycolysis. To our knowledge, the current experiments represent the first such examination of mildronate’s effect on oxidative metabolism, glycolysis and the pentose phosphate pathway. We have previously noted that mildronate pretreatment increased flux through pyruvate dehydrogenase (in vivo) for the myocardium, replicating the current conditions, and supporting our current observations [12]. We cannot exclude the potential for ‘off-target’ effects of mildronate not related to the inhibition of carnitine synthesis including increased plasma HDL concentrations and decreased fatty acid and β-hydroxybutyrate concentrations [22] and increased thyroid hormone production [77].

Early experiments demonstrated that STZ diabetes was associated with relative energy deprivation in the brain, manifesting as decreases in phosphocreatine, ATP and the ATP/ADP ratio [78], and this may reflect the growing evidence supporting mitochondrial oxidative damage associated with STZ diabetes [79]. We measured increased abundance of glycolytic metabolites including glucose-6-phosphate, fructose-6-phosphate and glyceraldehyde-3-phosphate, hinting at increased uptake of glucose facilitated by the low-Km glucose transporters [79] coupled with the high plasma glucose concentration that we record. For the brain, STZ diabetes decreased cortical expression of glycolytic enzymes including hexokinase, glyceraldehyde-3-phosphate and phosphofructokinase [80]. We postulate that this decreasing glycolytic enzyme expression may reflect a mechanism to decrease flux through glycolysis despite the increased abundance of the primary substrate, glucose.

## 5. Conclusions

We demonstrate the potential for mildronate treatment to blunt the rise in blood glucose noted for STZ diabetes. This was a consequence of decreased plasma and tissue carnitine concentrations, hence triggering a shift in metabolism from fatty acid to glucose oxidation. For liver tissue, mildronate restored metabolite abundance for the TCA cycle and, in part, decreased the reliance on anaplerosis to support the TCA cycle. By contrast, mildronate had no effect on brain TCA metabolite abundance, emphasising the obligate nature of glucose use in the brain, with limited capacity for mitochondrial ꞵ-oxidation. However, the increased uptake of glucose by the brain facilitated the increased oxidation of glucose to sorbitol mediated through augmented NADPH production, addressed by the pentose phosphate pathway. These data support the use of metabolic inhibitors like mildronate, as an addition to current therapies, to restore blood glucose in diabetic subjects, and we document only modest effects of other metabolic pathways.

## Figures and Tables

**Table 1:** Plasma metabolite concentrations for untreated and STZ-diabetic rats treated with meldonium (100 mg/kg/day). Data represents mean ± SD for *n* = 12 control and *n* = 6 treated rats. Meldonium and acyl-carnitines were measured with reference to standard curve quantified by Hydrophobic-Interaction Liquid Chromatography (HILIC) exploiting authenticated standard compounds. For further details, see Methods. Statistical significance: effect of mildronate—* *p* < 0.05, ** *p* < 0.01 and *** *p* < 0.001; effect of STZ diabetes—^+^ *p* < 0.05, ^++^ *p* < 0.01 and ^+++^ *p* < 0.001. ND not detected.

Metabolites [μg/mL]	Untreated		STZ Diabetes	
	Citrate Buffer	Mildronate	Citrate Buffer	Mildronate
Plasma mildronate [µg/mL]	0.54 ± 0.33	40 ± 0.17 ***	0.55 ± 0.17	32 ± 2.2 ***
Liver mildronate [µg/gm]	ND	213 ± 37 ***	ND	194 ± 39 ***
Brain mildronate [µg/gm]	ND	281 ± 61 ***	ND	182 ± 23 ***
Insulin (ng/mL)	0.27 ± 0.12	0.29 ± 0.11	0.08 ± 0.04 ^+^	0.06 ± 0.05 ^+^
Plasma glucose (mM)	9.37 ± 2.69	9.26 ± 1.90	15.23 ± 1.55 ^+++^	10.92 ± 1.80 ^+++^*
l-Carnitine	1.11 ± 0.66	0.55 ± 0.14 **	0.65 ± 0.19 ^+^	0.32 ± 0.05 **^+^
Acetyl-carnitine	3.4 ± 1.8	15 ± 21	1.8 ± 0.090	2.8 ± 6.0
C3-acyl-carnitine	0.20 ± 0.057	0.052 ± 0.030 ***	0.12 ± 0.025 ^++^	0.034 ± 0.034 ^++^***
C5-acyl-carnitine	0.11 ± 0.028	0.11 ± 0.014	0.10 ± 0.019	0.11 ± 0.014
C6-acyl-carnitine	12 ± 2.7	3.6 ± 1.8 ***	5.2 ± 1.4 ^+++^	2.6 ± 2.1 ^++^***
C14-acyl-carnitine	6.9 ± 3.7	1.4 ± 0.74 ***	3.5 ± 1.4 ^+^	0.75 ± 0.55 ^+^***
C18-acyl-carnitine	2.8 ± 2.5	1.0 ± 0.45 *	1.7 ± 0.64	1.0 ± 0.75 *
C18:1-acyl-carnitine	1.4 ± 1.02	0.82 ± 0.43 *	1.7 ± 0.37	0.67 ± 0.30 *

**Table 2:** Liver metabolite concentrations for untreated and STZ-diabetic rats treated with meldonium (100 mg/kg/day). Data represents mean ± SD for *n* = 12 control and *n* = 6 treated rats. Carnitine and acyl-carnitines represent fold change compared with untreated rats and were measured with reference to standard curve quantified by Hydrophobic-Interaction Liquid Chromatography (HILIC) exploiting authenticated standard compounds. For further details, see Methods. Statistical significance: effect of mildronate—*** *p* < 0.001; effect of STZ diabetes—^++^ *p* < 0.01.

Metabolites [Fold Change]	Untreated		STZ-Diabetes	
	Citrate Buffer	Mildronate	Citrate Buffer	Mildronate
2-Methylbutyroylcarnitine	1.0 ± 1.38	0.27 ± 0.50	1.34 ± 3.11	0.06 ± 0.02
3-Dehydroxycarnitine	1.0± 0.44	0.93 ± 0.29	1.08 ± 0.53	1.77 ± 0.69
Butyryl-carnitine	1.0 ± 0.41	0.41 ± 0.17 ***	0.71 ± 0.24	0.36 ± 0.22 ***
Coenzyme A	1.0 ± 0.13	0.92 ± 0.08	1.07 ± 0.51	1.64 ± 0.57 ^++^
Dodecanoyl-carnitine	1.0 ± 1.53	0.11 ± 0.24	0.24 ± 0.59	0.01 ± 0.01
Acetyl-carnitine	1.0 ± 2.92	0.18 ± 0.44	1.08 ± 2.65	0 ± 0
Carnitine	1.0 ± 0.34	0.49 ± 0.07 ***	0.72 ± 0.31	0.46 ± 0.01 ***
Hexanoyl-carnitine	1.0 ± 1.34	0.97 ± 1.62	0.68 ± 0.95	0.35 ± 0.15
Methylmalonyl-carnitine	1.0 ± 0.98	0.69 ± 1.70	0.95 ± 1.24	0.17 ± 0.35
Decanoyl-L-carnitine	1.0 ± 0.88	1.40 ± 0.24	1.49 ± 0.27	1.53 ± 0.18
Oleoylcarnitine	1.0 ± 0.93	0.58 ± 0.25	0.57 ± 0.48	0.53 ± 0.12
Stearoyl-carnitine	1.0 ± 1.74	0.36 ± 0.80	0.23 ± 0.47	0.02 ± 0.03

**Table 3:** Brain metabolite concentrations for untreated and STZ-diabetic rats treated with meldonium (100 mg/kg/day). Data represents mean ± SD for *n* = 12 control and *n* = 6 treated rats. Carnitine and acyl-carnitines represent fold change compared with untreated rats and were measured with reference to standard curve quantified by Hydrophobic-Interaction Liquid Chromatography (HILIC) exploiting authenticated standard compounds. For further details, see Methods. Statistical significance: effect of mildronate—* *p* < 0.05, ** *p* < 0.01 and *** *p* < 0.001; effect of STZ diabetes—^+^ *p* < 0.05.

Metabolites [Fold Change]	Untreated		STZ-Diabetes	
	Citrate Buffer	Mildronate	Citrate Buffer	Mildronate
2-Methylbutyroylcarnitine	1.0 ± 0.44	2.16 ± 1.34	1.57 ± 1.72	1.69 ± 0.46
3-Dehydroxycarnitine	1.0 ± 0.58	0.67 ± 0.58	1.53 ± 0.99	0.88 ± 0.39
3-Hydroxybutyryl-carnitine	1.0 ± 1.01	0.03 ± 0.05 **	0.57 ± 0.66	0.16 ± 0.19 **
Decanoyl-L-carnitine	1.0 ± 0.40	0.90 ± 0.13	1.26 ± 0.87	1.73 ± 2.10
Dodecanoyl-carnitine	1.0 ± 0.81	0.55 ± 0.67	0.89 ± 0.84	0.69 ± 0.51
Acetyl-carnitine	1.0 ± 0.62	0.24 ± 0.13 *	1.29 ± 1.71	0.33 ± 0.39 *
Carnitine	1.0 ± 0.32	0.29 ± 0.16 ***	0.60 ± 0.20 ^+^	0.34 ± 0.06 ^+^***
Hexanoyl-carnitine	1.0 ± 0.51	0.26 ± 0.19 ***	1.03 ± 0.67	0.36 ± 0.12 ***
Palmitoylcarnitine	1.0 ± 0.52	0.53 ± 0.76	0.48 ± 0.32	0.43 ± 0.34
Methylmalonyl-carnitine	1.0 ± 0.74	0.43 ± 0.63	0.34 ± 0.49 ^+^	0.28 ± 0.17 ^+^
Octanoyl-carnitine	1.0 ± 0.46	0.43 ± 0.66 ***	2.15 ± 0.42 ^+^	0.57 ± 0.45 ***
Stearoyl-carnitine	1.0 ± 0.63	0.31 ± 0.29 **	0.56 ± 0.63	0.19 ± 0.18 **
Tetradecanoyl-carnitine	1.0 ± 0.55	0.41 ± 0.48 *	0.66 ± 0.41	0.58 ± 0.33

## Data Availability

The original contributions presented in this study are included in the article and Supplementary Materials. Further inquiries can be directed to the corresponding author.

## Supplementary Materials

The following supporting information can be downloaded at: https://www.mdpi.com/article/10.3390/metabo16010061/s1. Table S1: Number and classification of ion features isolated by chromatography-mass spectrometry for each tissue by each respective method of analysis; Table S2: Estimation of Accuracy, R^2^, Q^2^ and permutation analysis for Partial Least Squares Discrimination Analysis (PLS-DA) following 4 chromatographic methods for untargeted ion features from plasma, liver and brain samples. Ion features data was imported into MetaboAnalyst 6.0; Table S3: 2-Factor between subject ANOVA for plasma samples from STZ-diabetes or mildronate-treated rats; Table S4: 2-Factor between subject ANOVA for liver samples from STZ-diabetes or mildronate-treated rats; Table S5: 2-Factor between subject ANOVA for brain samples from STZ-diabetes or mildronate-treated rats; Figure S1: Principal component multivariate analysis for all ion features detected by ion Exchange (i), C18-reverse phase chromatography (ii); derivatised-C18 reverse phase Chromatography mass spectrometry (iii) and Hydrophobic Interaction Liquid Chromatography (HILIC)-mass spectrometry (iv) for plasma samples [A], Liver [B] and brain [C] tissue from untreated and STZ-diabetic rats supplemented with mildronate; Figure S2: Correlation analysis for metabolites in brain tissue. Data S1: Brain-Raw Data; Data S2: Liver-Raw Data, Data S3: Plasma-Raw Data.
