# Supplementary material for: Changes in the Metabolome of Different Tissues in Response to Streptozotocin Diabetes and Mildronate Exposure: A Metabolomic Assessment
Source: Metabolites. 2026 Jan 9;16(1):61. doi: 10.3390/metabo16010061 (PMC12843979; doi:10.3390/metabo16010061)
Supplement: Supplementary file 1 [file metabolites-16-00061-s001.zip › SUPPLEMENTAL DATA-STZ&Mildronate-Nov2025.pdf]

SUPPLEMENTAL DATA

Table S1: Number and classification of ion features isolated by chromatography-mass spectrometry for each tissue by each respective method of analysis.

Tissue & Methods	Total ion features	Ion features CV<30%	Identified compounds
PLASMA			
Ion Exchange	4142	1670	270
C18-Reverse Phase	10681	2650	242
Derivatised-C18-Reverse Phase	6725	4729	91
Hydrophobic Interaction Liquid Chromatography	13996	2648	127
LIVER			
Ion Exchange	6646	1954	239
C18-Reverse Phase	6287	1928	118
Derivatised-C18-Reverse Phase	6733	4124	131
Hydrophobic Interaction Liquid Chromatography	13996	362	127
BRAIN			
Ion Exchange	6071	2008	119
C18-Reverse Phase	6287	2259	101
Derivatised-C18-Reverse Phase	5619	4081	84
Hydrophobic Interaction Liquid Chromatography	13996	1819	127

TABLE S2: Estimation of Accuracy, R2, Q2 and permutation analysis for Partial Least Squares Discrimination Analysis (PLS-DA) following 4 chromatographic methods for untargeted ion features from plasma, liver and brain samples. Ion features data was imported into MetaboAnalyst 6.0. Data was filtered by interquartile range, and normalised and scaled (mean-centring/SD).

	Chromatographic Methods			
**Measurement**	**Ion Exchange**	**C18-Reverse Phase**	**Derivatised C18 (AAA)**	**Hydrophobic interaction liquid chromatography**
Plasma				
Accuracy	0.68	0.35	0.573	0.56
Q^2	0.999	0.999	0.998	0.999
R^2	-0.47	-0.43	-0.12	-0.133
PLS-DA Permutation Analysis	0.002	0.996	0.037	0.387
Liver				
Accuracy	0.927	0.938	0.893	0.353
Q^2	0.999	0.999	0.999	0.998
R^2	0.902	0.599	0.515	0.194
PLS-DA Permutation Analysis	0.346	0.299	0.631	0.183
Brain				
Accuracy	0.838	0.633	0.817	0.787
Q^2	0.999	0.999	0.999	0.999
R^2	0.757	0.212	0.502	0.445
PLS-DA Permutation Analysis	0.212	0.268	0.482	0.114

Table S3: 2-Factor between subject ANOVA for plasma samples from STZ-diabetes or mildronate-treated rats.

**Identified compound**	STZ (F.val)	STZ (raw.p)	STZ (adj.p)	Mildronate (F.val)	Mildronate (raw.p)	Mildronate (adj.p)	Interaction (F.val)	Interaction (raw.p)	Interaction (adj.p)
3-hydroxymethyl-glutarate	411.5	8.24E-15	1.65E-12	3.72	0.068	0.38857	0.107	0.747	0.98854
Oxoadipic acid	361.76	2.81E-14	2.81E-12	1.811	0.193	0.58485	0.054	0.818	0.98854
Glutaric acid	320.7	8.80E-14	5.10E-12	0.795	0.383	0.76415	0.089	0.769	0.98854
Gluconolactone	315.69	1.02E-13	5.10E-12	1.969	0.176	0.57705	0.556	0.465	0.90291
Glucose	261.97	5.87E-13	2.35E-11	3.991	0.06	0.36364	1.756	0.2	0.80351
Galacturonic acid	213.62	3.88E-12	1.29E-10	0.316	0.58	0.87879	0.002	0.966	0.98995
Quinolinic acid	207.08	5.16E-12	1.47E-10	2.779	0.111	0.46939	3.917	0.062	0.71579
1-Deoxy-D-xylulose-5-phosphate	168.1	3.42E-11	8.55E-10	0.102	0.753	0.92832	0.34	0.567	0.945
Ribose	165.42	3.96E-11	8.80E-10	2.778	0.111	0.46939	2.192	0.154	0.79524
5-Hydroxyindoleacetic acid	146.68	1.16E-10	2.32E-09	2.6	0.123	0.47547	2.46	0.132	0.79524
Cysteine-S-sulfate	121.83	5.87E-10	1.07E-08	3.06	0.096	0.46939	2.123	0.161	0.79524
Arginine	103.81	2.31E-09	3.85E-08	2.827	0.108	0.46939	3.396	0.08	0.78095
2-C-Methyl-D-Erythritol 4-phosphate	90.614	7.20E-09	1.11E-07	3.381	0.081	0.45	0.671	0.422	0.88571
Salicylic Acid	89.249	8.16E-09	1.13E-07	2.342	0.142	0.50714	1.552	0.227	0.80351
Deoxyadenosine	88.818	8.49E-09	1.13E-07	1.61	0.219	0.64058	1.259	0.275	0.82895
Malitol	79.339	2.13E-08	2.66E-07	1.622	0.217	0.64058	1.759	0.2	0.80351
Norophthalmic acid	68.438	6.92E-08	8.14E-07	0.831	0.373	0.76415	2.261	0.148	0.79524
Glycocholic acid	67.785	7.46E-08	8.29E-07	0.845	0.369	0.76415	4.286	0.052	0.71579
2,5 Dihydroxybenzoic Acid	64.105	1.15E-07	1.21E-06	4.095	0.057	0.36364	4.971	0.037	0.71579
3-Hydroxybutyric acid	59.766	1.97E-07	1.97E-06	8.193	0.01	0.13333	1.906	0.183	0.80351
Dehydro-quinic acid	54.16	4.13E-07	3.93E-06	0.004	0.953	0.98247	0.083	0.777	0.98854
L-Homoserine	50.761	6.65E-07	6.05E-06	0.12	0.732	0.92832	0.465	0.503	0.92696
Homocysteinesulfinic acid	50.239	7.17E-07	6.23E-06	0.099	0.756	0.92832	0.034	0.856	0.98854
Guanosine monophosphate	48.618	9.10E-07	7.58E-06	0.272	0.608	0.88986	0.227	0.639	0.98045
L-4-Hydroxyglutamate semialdehyde	46.407	1.27E-06	1.02E-05	0.006	0.938	0.98247	0.000409	0.984	0.98995
Ascorbic acid-2-sulfate	45.467	1.47E-06	1.13E-05	0.015	0.905	0.98247	0.051	0.823	0.98854
2-hydroxyglutarate	44.993	1.58E-06	1.17E-05	0.069	0.795	0.92832	1.081	0.311	0.82895
Creatinine	43.678	1.95E-06	1.39E-05	0.468	0.502	0.85085	1.156	0.295	0.82895
N-Acetyl-D-glucosamine	41.976	2.57E-06	1.77E-05	0.723	0.405	0.76415	0.275	0.606	0.98045
AMP	41.597	2.73E-06	1.82E-05	0.005	0.946	0.98247	0.004	0.949	0.98854
Neuraminic acid	39.414	3.95E-06	2.54E-05	1.567	0.225	0.64286	2.139	0.159	0.79524
Hippuric acid	39.258	4.06E-06	2.54E-05	1.967	0.176	0.57705	0.022	0.884	0.98854
Methyl beta-D-glucopyranoside	36.622	6.47E-06	3.92E-05	6.88	0.016	0.16	3.742	0.067	0.71579
Dopamine	35.984	7.26E-06	4.27E-05	2.954	0.101	0.46939	0.039	0.845	0.98854
Homovanillin	33.248	1.21E-05	6.91E-05	1.189	0.289	0.7225	0.755	0.395	0.88571
Glycerol 3-phosphate	29.526	2.56E-05	0.000142	0.016	0.9	0.98247	0.194	0.665	0.98382
Mannitol	29.388	2.63E-05	0.000142	7.403	0.013	0.13684	2.06	0.167	0.79524
Leucine	29.145	2.77E-05	0.000146	2.761	0.112	0.46939	0.008	0.929	0.98854
N-Acetyl-ornithine	28.85	2.95E-05	0.000151	4.489	0.047	0.36154	1.582	0.223	0.80351
(5R)-5-Hydroxyhexanoic acid	28.643	3.08E-05	0.000151	1.046	0.319	0.74943	1.419	0.248	0.82667
3-Methyladipic acid	28.605	3.10E-05	0.000151	0.401	0.534	0.87541	0.005	0.947	0.98854
5-Hydroxypentanoic acid	28.303	3.31E-05	0.000154	0.441	0.514	0.86387	0.027	0.871	0.98854
D-Phenyllactic acid	27.635	3.82E-05	0.000174	0.263	0.614	0.88986	0.665	0.425	0.88571
Beta-Guanidinopropionic acid	27.284	4.12E-05	0.000183	6.282	0.021	0.2	7.255	0.014	0.4

Serine	26.514	4.88E-05	0.000212	0.243	0.627	0.89189	1.106	0.306	0.82895
ADP	24.919	7.00E-05	0.000297	0.004	0.951	0.98247	0.095	0.761	0.98854
pyroglutamine	24.849	7.12E-05	0.000297	0.905	0.353	0.76415	2.738	0.114	0.79524
Phosphorylcholine Chloride	24.554	7.62E-05	0.000311	0.496	0.489	0.8431	0.683	0.418	0.88571
UTP	24	8.67E-05	0.000343	2.554	0.126	0.47547	0.97	0.336	0.85063
Indole-3-acetaldehyde	23.968	8.74E-05	0.000343	0.072	0.792	0.92832	0.34	0.567	0.945
Palmitoleic Acid	23.327	0.000102	0.000392	0.066	0.8	0.92832	0.066	0.8	0.98854
Indoxyl sulfate	22.497	0.000124	0.000468	2.494	0.13	0.48148	0.21	0.652	0.98045
Tyrosol	22.326	0.00013	0.000481	4.141	0.055	0.36364	0.041	0.842	0.98854
Aminocaproic acid	21.912	0.000144	0.000524	5.533	0.029	0.25217	2.422	0.135	0.79524
Glutamic acid	21.165	0.000173	0.000618	2.164	0.157	0.54828	2.063	0.166	0.79524
3-Oxodecanoic acid	21.075	0.000177	0.000621	2.719	0.115	0.46939	0.07	0.793	0.98854
L-Hexanoylcarnitine	20.894	0.000185	0.000638	42.012	2.55E-06	0.00017	11.694	0.003	0.2
Tyrosine	20.644	0.000198	0.000671	0.071	0.792	0.92832	0.019	0.891	0.98854
Gamma-aminobutyric acid	19.892	0.00024	8.00E-04	0.266	0.612	0.88986	0.000381	0.985	0.98995
Inosine monophosphate	19.817	0.000245	0.000803	2.42	0.135	0.49091	1.549	0.228	0.80351
1,2 Dihydroxybenzene	19.431	0.000271	0.000874	0.904	0.353	0.76415	0.074	0.789	0.98854
N-Acetyl-DL-Glutamic acid	19.026	0.000302	0.000959	5.677	0.027	0.24545	3.127	0.092	0.79167
2-Hydroxycinnamic acid	18.903	0.000312	0.000975	0.103	0.752	0.92832	0.155	0.698	0.98854
dGMP	18.4	0.000358	0.001102	0.002	0.962	0.98667	0.032	0.859	0.98854
N-Acetylleucine	18.33	0.000364	0.001103	1.92	0.181	0.57778	0.248	0.624	0.98045
Taurodeoxycholic acid	18.099	0.000388	0.001158	0.203	0.657	0.89189	3.996	0.059	0.71579
Kynurenine	17.623	0.000443	0.001303	0.477	0.498	0.85085	0.077	0.784	0.98854
Glucose 1-phosphate	17.194	0.000499	0.001446	0.138	0.715	0.92258	0.014	0.906	0.98854
Norvaline	16.99	0.000529	0.00151	1.858	0.188	0.58485	0.031	0.861	0.98854
O-Phospho-L-serine	16.942	0.000536	0.00151	0.811	0.379	0.76415	1.096	0.308	0.82895
Ribulose 5-phosphate	16.513	0.000606	0.001683	0.05	0.825	0.94286	0.005	0.944	0.98854
N-Formyl-L-methionine	16.385	0.000629	0.001703	0.403	0.533	0.87541	0.283	0.6	0.98045
Quinic acid	16.379	0.00063	0.001703	0.682	0.419	0.77593	0.007	0.933	0.98854
Methylmalonylcarnitine	16.171	0.000669	0.001784	3.015	0.098	0.46939	1.537	0.229	0.80351
Glucose-6-phosphate	15.75	0.000757	0.001992	0.203	0.657	0.89189	0.645	0.431	0.88571
Isobutyryl-L-carnitine	15.679	0.000773	0.002008	12.963	0.002	0.04	8.164	0.01	0.33333
Homocysteine	14.601	0.001	0.00241	0.038	0.848	0.9573	0.27	0.609	0.98045
Indole-3-lactic acid	13.521	0.001	0.00241	0.081	0.778	0.92832	0.235	0.633	0.98045
Jasmonic acid	13.628	0.001	0.00241	14.862	0.000987	0.0282	2.864	0.106	0.79524
Succinyl-Homoserine	14.327	0.001	0.00241	1.146	0.297	0.73333	0.027	0.87	0.98854
Taurocholic acid	14.757	0.001	0.00241	0.01	0.923	0.98247	1.195	0.287	0.82895
UMP	14.36	0.001	0.00241	0.045	0.833	0.94659	0.001	0.973	0.98995
Acetylglycine	12.009	0.002	0.004598	0.099	0.757	0.92832	0.983	0.333	0.85063
ADP-ribose	13.468	0.002	0.004598	0.347	0.562	0.87879	2.146	0.158	0.79524
CMP	12.841	0.002	0.004598	0.004	0.948	0.98247	0.043	0.838	0.98854
Proline	13.244	0.002	0.004598	8.741	0.008	0.12308	3.358	0.082	0.78095
Phosphoglycolic acid	11.543	0.003	0.006742	0.323	0.576	0.87879	0.009	0.925	0.98854
Ribose 5-phosphate	11.338	0.003	0.006742	0.01	0.922	0.98247	0.000594	0.981	0.98995
6-Hydroxy-5-methoxyindole glucuronide	10.648	0.004	0.008791	0.537	0.472	0.8431	1.356	0.258	0.82895
Pyridoxal 5'-phosphate	10.316	0.004	0.008791	0.339	0.567	0.87879	0.007	0.934	0.98854
Adipate semialdehyde	10.185	0.005	0.010417	0.499	0.488	0.8431	0.156	0.697	0.98854
Dimethyl fumarate	10.033	0.005	0.010417	0.527	0.476	0.8431	0.226	0.639	0.98045
Hypotaurine	9.733	0.005	0.010417	0.008	0.931	0.98247	3.716	0.068	0.71579
Lysine	9.93	0.005	0.010417	0.159	0.694	0.9098	0.416	0.526	0.92696

Propionyl-Carnitine	9.685	0.005	0.010417	60.41	1.82E-07	1.82E-05	3.929	0.061	0.71579
Phosphoenolpyruvic acid	9.349	0.006	0.012371	0.01	0.921	0.98247	0.009	0.927	0.98854
2-Isopropylmalic acid	8.919	0.007	0.014	0.199	0.66	0.89189	0.069	0.796	0.98854
Fructose 6-phosphate	9.216	0.007	0.014	0.09	0.767	0.92832	0.012	0.912	0.98854
Oleoylcarnitine	9.052	0.007	0.014	0.626	0.438	0.79636	1.582	0.223	0.80351
Acetylcysteine	8.671	0.008	0.015385	1.261	0.275	0.71429	2.165	0.157	0.79524
Isoleucine	8.7	0.008	0.015385	1.815	0.193	0.58485	0.004	0.949	0.98854
Mildronate	8.602	0.008	0.015385	68.735	6.69E-08	1.34E-05	8.52	0.008	0.32
N-Acetyl-L-methionine	8.518	0.008	0.015385	12.096	0.002	0.04	4.659	0.043	0.71579
Phenylpyruvic acid	8.498	0.009	0.017143	0.231	0.636	0.89189	2.054	0.167	0.79524
2-Phosphoglyceric acid	8.223	0.01	0.018519	0.253	0.62	0.89189	0.19	0.668	0.98382
S-Cysteinosuccinic acid	8.035	0.01	0.018519	0.083	0.776	0.92832	0.011	0.918	0.98854
UDP	8.163	0.01	0.018519	0.767	0.392	0.76415	0.075	0.787	0.98854
4-Hydroxyphenylpyruvic acid	7.922	0.011	0.02	2.135	0.159	0.54828	2.871	0.106	0.79524
N-acetyl-glucosamine-1-phosphate	7.787	0.011	0.02	0.051	0.823	0.94286	0.035	0.853	0.98854
Homogentisic acid	7.607	0.012	0.021622	1.014	0.326	0.74943	0.78	0.388	0.88571
2-Methylbutyroylcarnitine	6.791	0.017	0.030357	28.673	3.06E-05	0.00153	1.545	0.228	0.80351
Adipic acid	6.596	0.018	0.031304	5.035	0.036	0.288	0.721	0.406	0.88571
Guanosine	6.582	0.018	0.031304	0.881	0.359	0.76415	0.017	0.897	0.98854
Kynurenic acid	6.603	0.018	0.031304	0.157	0.696	0.9098	0.447	0.511	0.92696
deoxythymidine	6.408	0.02	0.034483	0.755	0.395	0.76415	0.368	0.551	0.94359
Tetradecanoylcarnitine	6.128	0.022	0.037607	24.663	7.43E-05	0.002972	2.796	0.11	0.79524
Methylisocitric acid	5.873	0.025	0.042017	0.024	0.879	0.97667	0.15	0.703	0.98854
Sarcosine	5.848	0.025	0.042017	1.214	0.284	0.7225	3.899	0.062	0.71579
Lactose	5.785	0.026	0.042975	1.105	0.306	0.73735	0.455	0.507	0.92696
L-Carnitine	5.745	0.026	0.042975	9.512	0.006	0.1	0.692	0.415	0.88571
Flavin Mononucleotide (unconfirmed)	5.621	0.028	0.045902	0.911	0.351	0.76415	3.073	0.095	0.79167
2-Oxoglutaric acid	5.431	0.03	0.04878	7.542	0.012	0.13333	0.694	0.415	0.88571
Sedoheptulose 7-phosphate	5.411	0.031	0.05	0.012	0.914	0.98247	0.599	0.448	0.88713
Octanoyl Carnitine	4.118	0.056	0.085496	19.319	0.000279	0.0093	10.622	0.004	0.2
Fumaric Acid	0.272	0.608	0.66448	12.196	0.002	0.04	0.158	0.695	0.98854

Table S4: 2-Factor between subject ANOVA for liver samples from STZ-diabetes or mildronate-treated rats.

**Identified compound**	STZ (F.val)	STZ (raw.p)	STZ (adj.p)	Mildronate (F.val)	Mildronate (raw.p)	Mildronate (adj.p)	Interaction (F.val)	Interaction (raw.p)	Interaction (adj.p)
Myricetin	82.062	3.27E-09	6.54E-07	0.796	0.381	0.6562	0.395	0.536	0.716
Saccharic acid	71.654	1.14E-08	1.14E-06	0.002	0.963	0.9797	1.711	0.203	0.60435
Glycocholic acid	59.332	6.13E-08	3.98E-06	6.708	0.016	0.15238	17.445	0.000337	0.0337
beta-hydroxy-isobutyric acid	57.56	7.96E-08	3.98E-06	3.484	0.074	0.38947	0.671	0.421	0.64211
Dehydro-quinic acid	50.096	2.57E-07	9.43E-06	1.151	0.294	0.62178	0.028	0.869	0.91958
Citraconic acid	49.505	2.83E-07	9.43E-06	41.602	1.14E-06	7.60E-05	2.659	0.116	0.60308
2-Oxoglutaric acid	38.866	1.92E-06	5.49E-05	9.567	0.005	0.071429	0.238	0.63	0.80382
N-Acetyl-D-glucosamine	38.18	2.20E-06	5.50E-05	0.295	0.592	0.78158	0.198	0.66	0.82625
Quinolinic acid	30.529	1.11E-05	0.000247	3.836	0.062	0.35429	1.796	0.193	0.60308
Coenzyme A	26.128	3.13E-05	0.00062	0.26	0.615	0.79103	0.004	0.948	0.97231
Phosphoenolpyruvic acid	25.783	3.41E-05	0.00062	0.013	0.909	0.96702	0.393	0.537	0.716
Dimethyl fumarate	24.624	4.57E-05	0.000762	0.211	0.65	0.80864	0.748	0.396	0.64032
6-Phosphogluconic acid	23.235	6.56E-05	0.001009	0.57	0.458	0.67153	0.063	0.804	0.91271
Serine	22.46	8.06E-05	0.001151	1.194	0.285	0.62178	0.974	0.334	0.63333
dTDP	21.847	9.51E-05	0.001268	0.822	0.374	0.65043	0.106	0.748	0.87485
3-Hydroxybutyric acid	21.126	0.000116	0.00145	5.72	0.025	0.21739	2.562	0.123	0.60308
Hippuric acid	20.851	0.000125	0.001471	0.581	0.453	0.67153	0.951	0.339	0.63333
Pyridoxamine 5-phosphate	19.945	0.000161	0.001705	0.591	0.449	0.67153	0.713	0.407	0.64211
Histidine	19.932	0.000162	0.001705	2.593	0.12	0.44815	1.786	0.194	0.60308
N-Acetylglutamic acid	19.715	0.000172	0.00172	1.238	0.277	0.62178	0.056	0.815	0.91271
3-hydroxymethyl-glutarate	19.325	0.000193	0.001838	0.777	0.387	0.6562	1.023	0.322	0.63333
3-Amino-2-methylpropanoic acid	18.986	0.000213	0.001936	8.335	0.008	0.10667	2.091	0.161	0.60308
Valine	18.59	0.000239	0.002078	0.836	0.37	0.65043	3.856	0.061	0.55652
Glucuronic acid	17.238	0.000359	0.002992	0.644	0.43	0.67153	2.099	0.16	0.60308
Ketoglutaric Acid	16.931	0.000394	0.003152	18.593	0.000239	0.006829	3.994	0.057	0.55652
Glyceric acid	16.071	0.000515	0.003962	2.005	0.17	0.51515	0.81	0.377	0.63333
Xylulose	15.736	0.000573	0.004244	0.904	0.351	0.63818	2.526	0.125	0.60308
Hypoxanthine	14.151	0.00096	0.006061	0.441	0.513	0.70897	0.828	0.372	0.63333
Acetylcysteine	14.128	0.000967	0.006061	1.182	0.288	0.62178	0.129	0.722	0.87059
Glucose 6-phosphate	13.045	0.001	0.006061	0.343	0.564	0.76216	0.818	0.375	0.63333
Norvaline	13.245	0.001	0.006061	4.254	0.05	0.35429	1.032	0.32	0.63333
Taurocholic acid	13.407	0.001	0.006061	0.038	0.846	0.91957	0.002	0.961	0.9797
TMP	13.554	0.001	0.006061	13.885	0.001	0.02	9	0.006	0.4
Fumaric Acid	10.946	0.003	0.017143	0.578	0.454	0.67153	1.337	0.259	0.60435
pyroglutamine	11.037	0.003	0.017143	0.286	0.598	0.7817	1.003	0.327	0.63333
Hypotaurine	9.845	0.004	0.022222	0.01	0.92	0.97354	0.016	0.902	0.93472
Sedoheptulose 7-phosphate	9.587	0.005	0.027027	0.072	0.791	0.89379	0.251	0.621	0.80129
dUDP	9.25	0.006	0.030769	0.826	0.372	0.65043	0.113	0.74	0.87059
Glycerol 3-phosphate	9.078	0.006	0.030769	1.252	0.274	0.62178	0.294	0.592	0.76883
Glucosamine 6-phosphate	8.486	0.008	0.039024	1.312	0.263	0.62178	0.024	0.879	0.92042
Methionine sulphoxide	8.335	0.008	0.039024	2.084	0.162	0.5125	6.344	0.019	0.55652
Aminoheptanoic acid	4.199	0.052	0.15072	74.433	8.08E-09	1.62E-06	4.409	0.046	0.55652
Sorbitol	3.889	0.06	0.16	55.26	1.13E-07	1.13E-05	22.554	7.86E-05	0.01572

Methyl beta-D-glucopyranoside	2.786	0.108	0.22268	20.357	0.000144	0.0048	0.033	0.857	0.91658
Butyrylcarnitine	2.578	0.121	0.23495	17.065	0.000378	0.00945	1.008	0.325	0.63333
Orotic acid	0.993	0.329	0.44832	16.518	0.000448	0.009956	0.041	0.84	0.91658
Ornithine	0.528	0.474	0.60126	23.99	5.38E-05	0.002152	0.917	0.348	0.63333
Maleic acid	0.501	0.486	0.6075	25.214	3.94E-05	0.00197	0.755	0.394	0.64032

Table S5: 2-Factor between subject ANOVA for brain samples from STZ-diabetes or mildronate-treated rats.

	Diabetes (F.val)	Diabetes (raw.p)	Diabetes (adj.p)	Mildronate (F.val)	Mildronate (raw.p)	Mildronate (adj.p)	Interaction (F.val)	Interaction (raw.p)	Interaction (adj.p)
Sorbitol-6-phosphate	149.8	2.70E-12	5.40E-10	0.000835	0.977	1	0.018	0.895	0.93717
Sorbitol	119.33	3.26E-11	3.26E-09	0.412	0.527	0.88548	0.133	0.718	0.9037
Oxoadipic acid	91.263	5.44E-10	3.63E-08	0.054	0.819	0.93446	1.432	0.242	0.71176
Glucose-6-phosphate	52.964	9.95E-08	4.98E-06	4.282	0.049	0.5	3.002	0.095	0.60488
Taurine	44.699	4.30E-07	1.72E-05	0.639	0.431	0.83689	1.103	0.303	0.7439
Arabinonic acid	43.396	5.50E-07	1.83E-05	1.568	0.222	0.69394	2.324	0.139	0.6619
Gluconate	40.846	9.05E-07	2.50E-05	6.18E-05	0.994	1	0.522	0.476	0.82783
Glucose	40.33	1.00E-06	2.50E-05	0.089	0.768	0.9253	1.905	0.179	0.70196
Deoxyuridine	36.916	2.03E-06	4.36E-05	0.023	0.88	0.95978	0.42	0.523	0.84603
Inosinic acid/IMP	36.238	2.34E-06	4.36E-05	0.000264	0.987	1	0.061	0.808	0.92343
Phosphoglycolic acid	36.116	2.40E-06	4.36E-05	0.655	0.426	0.83689	0.027	0.871	0.93656
Xanthylate	32.573	5.26E-06	8.69E-05	1.58	0.22	0.69394	2.528	0.124	0.60488
Dihydroxyacetone	32.258	5.65E-06	8.69E-05	0.181	0.674	0.90759	1.917	0.178	0.70196
5-Hydroxyhexanoic acid	30.813	7.90E-06	0.000113	0.000902	0.976	1	0.124	0.727	0.9037
6-phosphono glucono-lactone	30.053	9.46E-06	0.000126	3.157	0.087	0.56875	2.576	0.121	0.60488
Histidine	26.771	2.12E-05	0.000265	2.07	0.162	0.63462	0.332	0.57	0.87692
Threonine	20.681	0.000111	0.001306	0.773	0.387	0.81474	2.644	0.116	0.60488
Glyceraldehyde 3-phosphate	19.952	0.000137	0.00142	1.356	0.255	0.69737	0.673	0.419	0.81468
1-Methyl Histidine	19.874	0.000141	0.00142	0.033	0.857	0.95222	0.007	0.933	0.95612
Homovanillic acid	19.834	0.000142	0.00142	0.146	0.706	0.90759	1.509	0.23	0.71176
dCMP	19.163	0.000174	0.001657	0.003	0.958	0.99792	0.372	0.547	0.85469
N-Acetyl-L-phenylalanine	18.97	0.000184	0.001673	0.059	0.81	0.93446	0.043	0.837	0.93656
Acetylphosphate	18.748	0.000197	0.001713	0.954	0.338	0.76818	5.575	0.026	0.48
Deoxyguanosine	18.004	0.000247	0.002058	0.012	0.912	0.96632	6.932	0.014	0.48
Hydrocinnamic acid	17.605	0.00028	0.00224	1.631	0.213	0.69394	1.762	0.196	0.7082
2-Aminobut-2-enoate	17.289	0.000309	0.002377	0.877	0.358	0.78681	4.079	0.054	0.57895
beta-hydroxy-isobutyric acid	15.059	0.000638	0.004726	0.892	0.354	0.78667	1.703	0.203	0.7082
Phenylalanine	14.446	0.000784	0.0056	0.276	0.604	0.90294	1.624	0.214	0.7082
Homoserine	13.928	0.000937	0.006219	1.332	0.259	0.69737	0.511	0.481	0.82931
1-Hydroxy-2-methyl-2-butenyl 4-pyrophosphate	13.872	0.000955	0.006219	0.273	0.606	0.90294	6.087	0.021	0.48
Mevalonic acid-5P	13.846	0.000964	0.006219	2.921	0.099	0.58235	0.018	0.893	0.93717
3-Hydroxybutyric acid	13.269	0.001	0.00625	6.541	0.017	0.22667	4.499	0.044	0.55
Sedoheptulose 1-phosphate	11.35	0.002	0.011765	2.653	0.115	0.60526	1.271	0.27	0.73867
Tyrosine	12.286	0.002	0.011765	2.428	0.131	0.63462	3.733	0.064	0.60488
5-Hydroxymethyl-2'-deoxyuridine	9.485	0.005	0.027778	1.453	0.239	0.69737	2.169	0.153	0.66522
Tryptophan	9.533	0.005	0.027778	0.103	0.751	0.91879	0.268	0.609	0.8922
N-Acetyl-DL-Valine	9.124	0.006	0.032432	11.802	0.002	0.08	2.813	0.106	0.60488
Pantothenic acid	8.393	0.008	0.042105	0.441	0.512	0.88548	0.15	0.702	0.9037
1-Amino-propan-2-ol	8.03	0.009	0.043902	3.69	0.066	0.52308	0.492	0.489	0.83361
Lysine	7.866	0.009	0.043902	1.345	0.257	0.69737	0.957	0.337	0.78372
Mucic Acid	7.935	0.009	0.043902	4.03	0.055	0.5	0.299	0.589	0.88571

6-Phosphogluconic acid	7.52	0.011	0.05	0.011	0.918	0.96632	0.55	0.465	0.82301
Isopentenyl pyrophosphate	7.51	0.011	0.05	0.247	0.623	0.90759	3.387	0.077	0.60488
pyroglutamine	7.409	0.011	0.05	0.358	0.555	0.888	1.741	0.199	0.7082
L-Carnitine	5.394	0.028	0.1037	33.001	4.77E-06	0.000477	6.023	0.021	0.48
Mildronate	3.7	0.065	0.20312	71.815	5.91E-09	1.18E-06	4.906	0.036	0.48
Beta-Guanidinopropionic acid	2.193	0.151	0.33556	14.436	0.000787	0.03935	0.358	0.555	0.86047
L-Hexanoylcarnitine	0.122	0.73	0.89693	17.129	0.000325	0.021667	0.041	0.84	0.93656

Supplement Figure 1

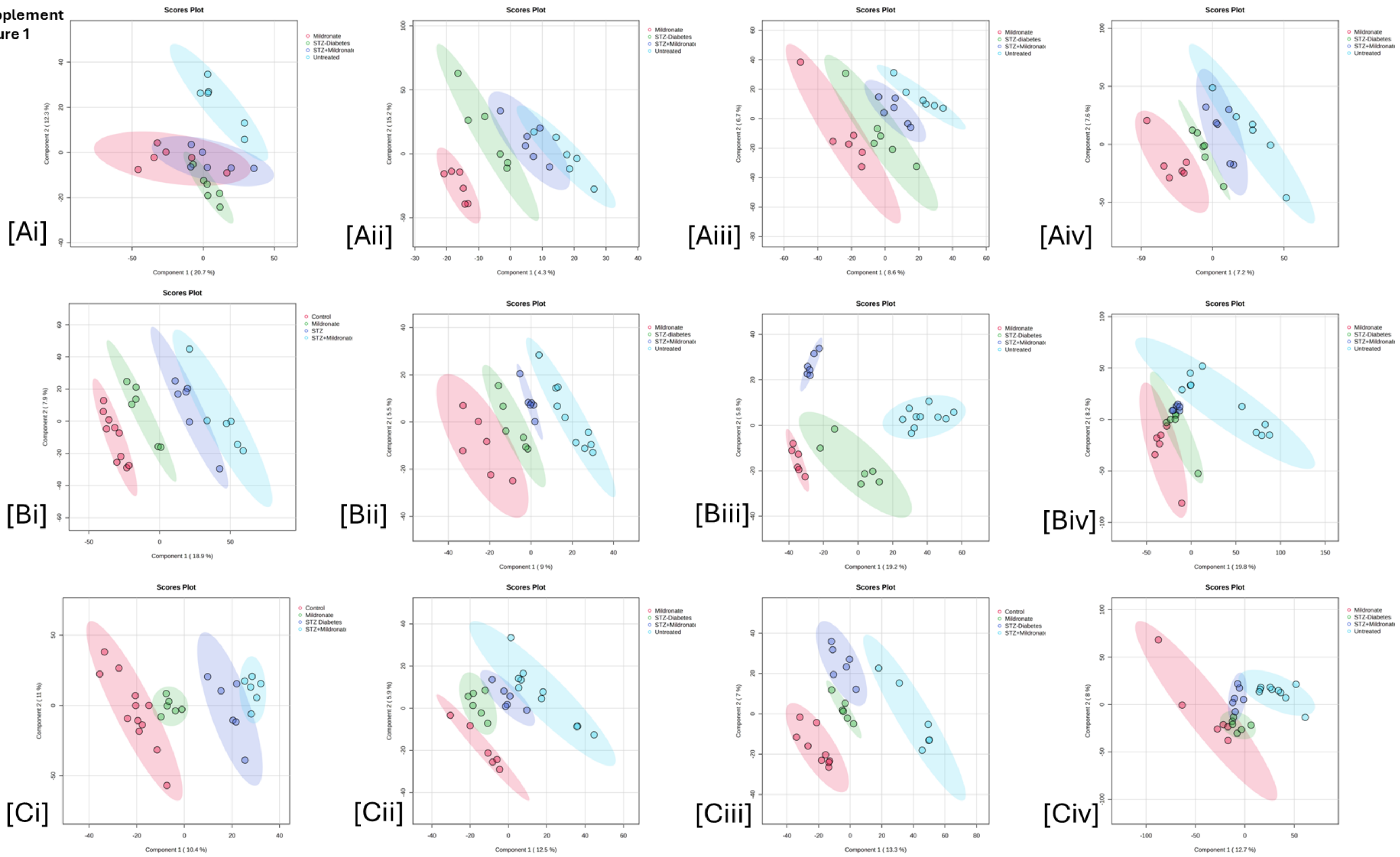

Supplemental Figure S1: Principal component multivariate analysis for all ion features detected by ion Exchange (i), C18-reverse phase chromatography (ii); derivatised-C18 reverse phase Chromatography-mass spectrometry (iii) and Hydrophobic Interaction Liquid Chromatography (HILIC)-mass spectrometry (iv) for plasma samples [A], Liver [B] and brain[C] tissue from untreated and STZ-diabetic rats supplemented with mildronate. Data represents Principal Component Analysis for all ion features detected. N=6-12 samples/group. Data was normalised by mean and analysis carried out in MetaboAnalyst6.0. For further details see methods.

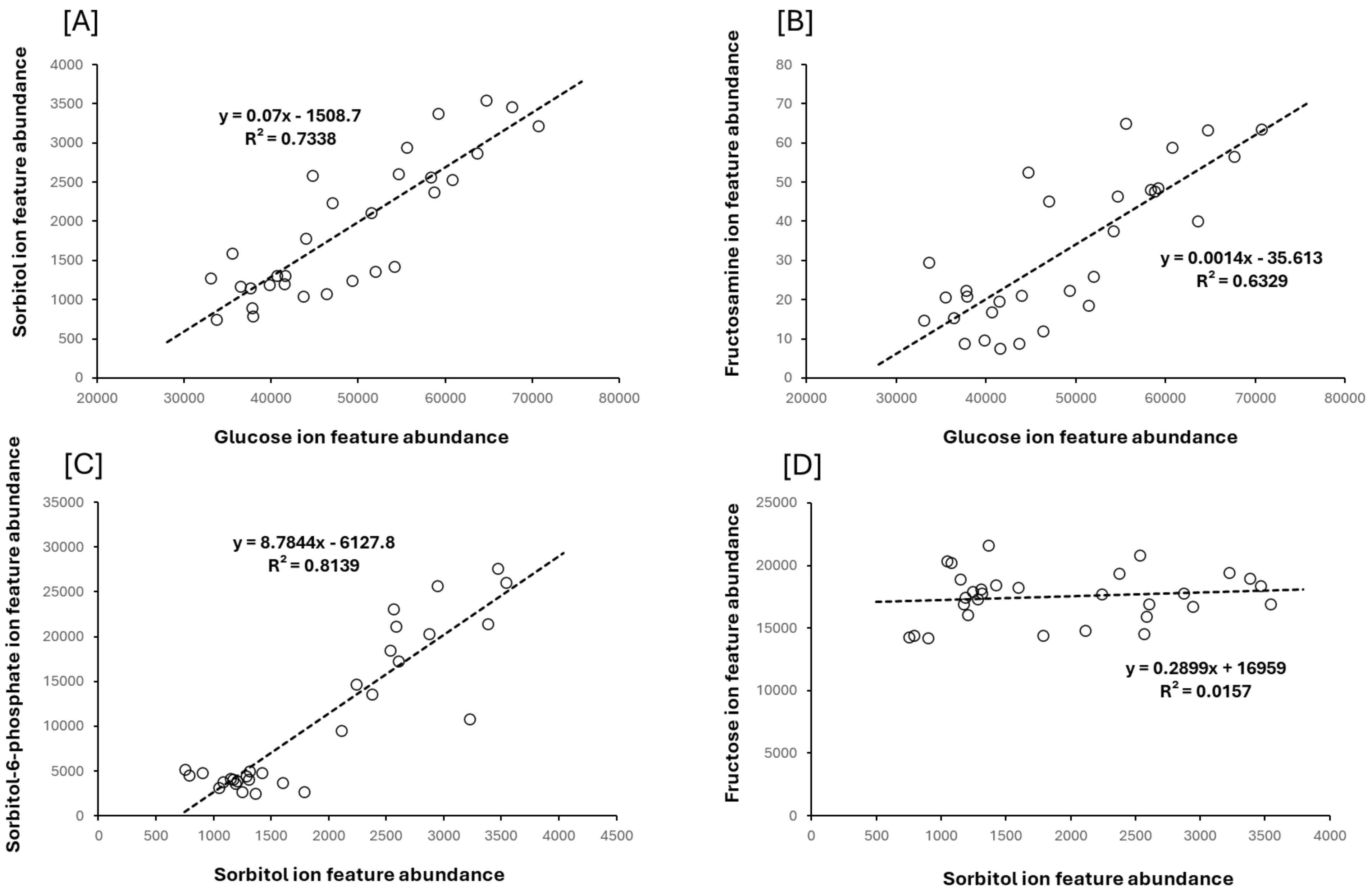
[A]
Sorbitol ion feature abundance
y = 0.07x - 1508.7
R2 = 0.7338
Glucose ion feature abundance
[B]
Fructosamine ion feature abundance
y = 0.0014x - 35.613
R2 = 0.6329
Glucose ion feature abundance
[C]
Sorbitol-6-phosphate ion feature abundance
y = 8.7844x - 6127.8
R2 = 0.8139
Sorbitol ion feature abundance
[D]
Fructose ion feature abundance
y = 0.2899x + 16959
R2 = 0.0157
Sorbitol ion feature abundance

Figure 2

Supplemental Figure S2.Correlation analysis for metabolites in brain tissue. Data represents ion intensities for identified metabolites in brain tissue showing the correlation between sorbitol and glucose [A], fructosamine and glucose [B], sorbitol-6-phosphate and sorbitol [C] and fructose and sorbitol [D]. Data represents ion features identified with reference to authenticated standards. N=6-12 samples/group. For further details see methods.
